# *Proctocaecum coronarium and Proctocaecum diploporum* (Digenea: Cryptogonimidae): Morphologically- and phylogenetically-distinct but closely related and sympatric crocodilian trematodes with identical life cycles

**DOI:** 10.1017/S0031182026102340

**Published:** 2026-06

**Authors:** Kamila Cajiao-Mora, Stephen S. Curran, John H. Brule, Daniel Gordillo-González, Haley R. Dutton, Micah B. Warren, Stephen A. Bullard

**Affiliations:** 1 Southeastern Cooperative Fish Parasite and Disease Laboratory and Southeastern Cooperative Fish Genetics Laboratory, School of Fisheries, Aquaculture, and Aquatic Sciences, College of Agriculture, Auburn University, Auburn, AL, USA; 2 Department of Zoology, School for Environmental Sciences and Development, North-West University, Private Bag X6001, Potchefstroom, South Africa

**Keywords:** Alabama, estuary, life history, Opisthorchioidea, reptile parasites, saltmarsh, South Carolina

## Abstract

During a parasitological survey of the cockscomb hydrobe, *Littoridinops monroensis* (Gastropoda: Cochliopidae), sympatric saltmarsh fishes and the American alligator, *Alligator mississippiensis* (Alligatoridae) within a saltmarsh pond in the Bon Secour Bay (Mobile Bay, Alabama, north-central Gulf of America) and other localities, we collected specimens of *Proctocaecum diploporum* (Stunkard, 1931) Baugh, 1957 and *Proctocaecum coronarium* (Cobbold, 1861) Brooks, 1980 (Cryptogonimidae). Fish scales were infected with 2 metacercarial cyst size classes, a small (<200 μm) and a large (>240 μm), suggesting the presence of distinct but closely related sympatric cryptogonimids. Morphological and nucleotide evidence demonstrated that *P. diploporum* (small cyst) and *P. coronarium* (large cyst), previously regarded as taxonomic synonyms, share the same first intermediate host (cockscomb hydrobe), are encysted within the scales of the same fish host species, and mature in the intestine of the same definitive host. The taxonomy of these trematodes and that of another similar American alligator trematode, *Caimanicola pavida* (Brooks and Overstreet, 1977) Brooks, 1980, needed reconsideration. Herein, we describe the first complete and naturally occurring life cycle of a reptile-infecting cryptogonimid, provide the first nucleotide data for a species of *Proctocaecum*, taxonomically reassess both *P. diploporum* and *P. coronarium*, and designate a neotype for *P. coronarium*. We consider *C. pavida* a junior subjective synonym of *P. coronarium*. Phylogenetically related and sympatric trematodes that use the same invertebrate and vertebrate hosts such as these *Proctocaecum* spp. could be a good model to further explore the mechanisms of speciation and host specificity among trematodes with complex life cycles.

## Graphical abstract

**Figure:**
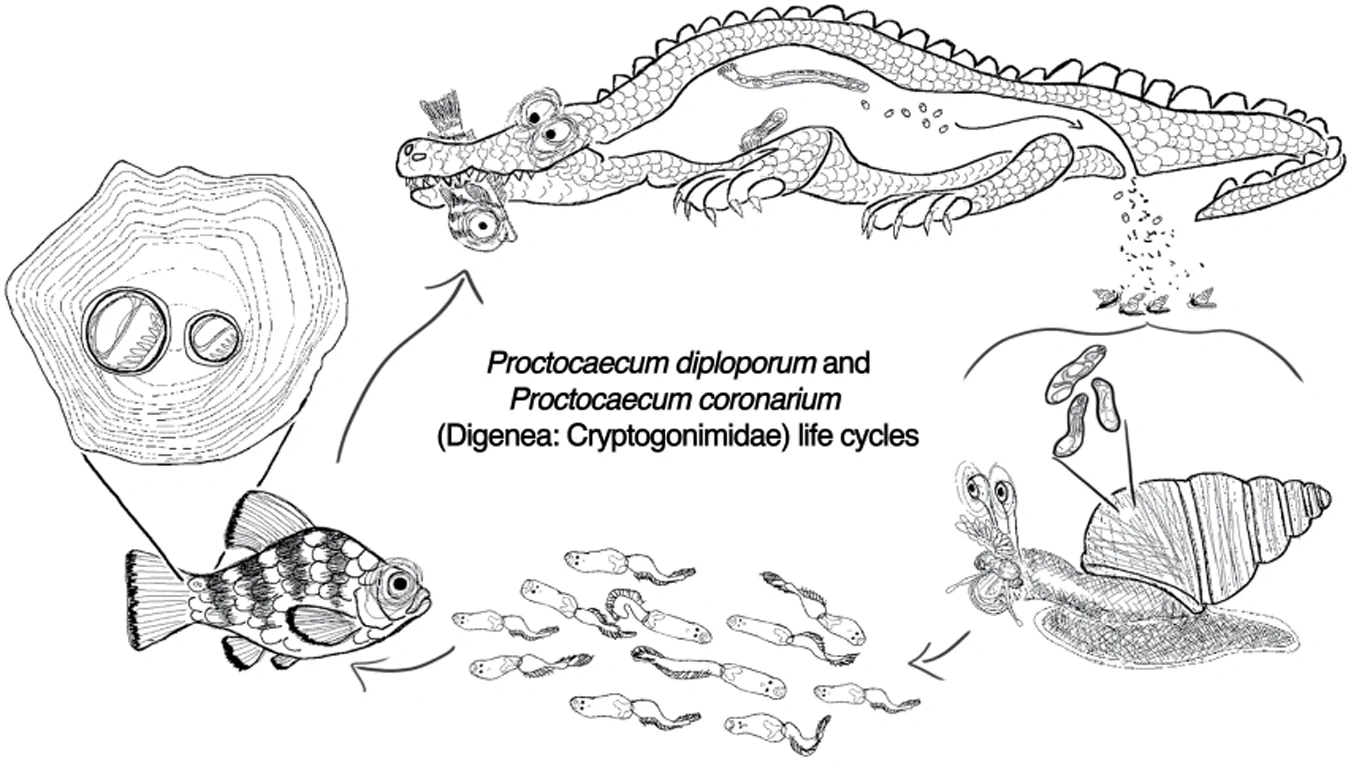

## Introduction

Cryptogonimidae Osborn, 1903 (Digenea: Opisthorchioidea) comprises >70 genera and 200 nominal species that infect poikilothermic vertebrates worldwide (Miller and Cribb, 2007, 2008a, 2008b, 2009, 2013; Miller et al., 2009; Kmentová et al., 2020; Montes et al., 2025). A vast majority of cryptogonimids mature in fishes (Miller and Cribb, 2008b), and only 7 accepted genera (∼10%) include species that infect amphibians and reptiles: *Andrincola* Zhang and Yang, 1993 and *Andritrema* Zhang and Yang, 1993 from amphibians in China (Zhan and Yang, 1993); *Proctocaecum* Baugh, 1957 and *Caimanicola* Teixeira de Freitas and Lent, 1938 from reptiles in the Americas, Africa, Australia and Asia (Brooks, 1980; Miller and Cribb, 2008b); *Timoniella* Rebecq, 1960, *Acanthostomum* Looss, 1899 and *Neochasmus* Van Cleave and Mueller, 1932, from fishes and reptiles in the Americas, Africa and Asia (Brooks, 1970; Miller and Cribb, 2008b). Relevant for the present study are the *Proctocaecum* species (Table 1). Specifically, cryptogonimids infecting the American alligator (*Alligator mississippiensis* [Daudin, 1802] [Alligatoridae]): *Proctocaecum diploporum* (Stunkard, 1931) Baugh, 1957 (type species), *Proctocaecum coronarium* (Cobbold, 1861) Brooks, 1980 and *Caimanicola pavida* (Brooks and Overstreet, 1977) Brooks, 1980.

**Table 1.** *Proctocaecum* Baugh, 1957 species with associated type host, type locality and museum type series Table 1 long description.

*Proctocaecum* spp.	Type host	Type locality	Type series	Reference
*Proctocaecum diploporum* (Stunkard, 1931) Baugh, 1957 (type)	American alligator, *Alligator mississippiensis* (Daudin, 1802) (Crocodilia: Alligatoridae)	Unknown	AMNH 875 (holotype), 875A (paratype)	Stunkard (1931); present study
*Proctocaecum coronarium* (Cobbold, 1861) Brooks, 1980	American alligator	Alabama, USA	USNM 1774187 (neotype)	Cobbold (1861); present study
*Proctocaecum productum* (Odhner, 1902) Brooks, 1980	Nile crocodile, *Crocodylus niloticus* Laurenti, 1768 (Crocodilia: Crocodylidae)	Sudan	–	Odhner (1902)
*Proctocaecum vicinum* (Odhner, 1902) Brooks, 1980	Nile crocodile	Sudan	–	Odhner (1902)
*Proctocaecum elongatum* (Tubangui and Masiluñgan, 1936) Brooks, 1980	Saltwater crocodile, *Crocodylus porosus* Schneider, 1801	Palawan, Philippines	Philippine Bureau of Science Parasitological Collection No. 474^a^	Tubangui and Masiluñgan (1936)
*Proctocaecum atae* (Tubangui and Masiluñgan, 1936) Brooks, 1980	Saltwater crocodile	Palawan, Philippines	Philippine Bureau of Science Parasitological Collection No. 500^a^	Tubangui and Masiluñgan (1936)
*Proctocaecum gonotyl* (Dollfus, 1950) Brooks, 1980	Nile crocodile	Democratic Republic of the Congo	–	Dollfus (1950)
*Proctocaecum crocodili* (Yamaguti, 1954) Baugh, 1957	Saltwater crocodile	Sulawesi, Indonesia	MPM 22348 (syntypes)	Yamaguti (1954)
*Proctocaecum nicolli* Brooks, 1980	Freshwater crocodile, *Crocodylus johnstoni* (Krefft, 1873)	North Queensland, Australia	USNM 74504 (holotype); USNM 75554 (paratype)^b^	Brooks (1980)
*Proctocaecum dorsale* Catto and Amato, 1993	Yacare caiman, *Caiman yacare* (Daudin, 1801) (Crocodilia: Alligatoridae)	Mato Grosso do Sul, Brazil	CHIOC 32896a (holotype); CHIOC 32896b-c, 32897a-b, 32898, 32899a-b, 33000a-b, USNM82629, 82630 (paratypes)	Catto and Amato (1993)
*Proctocaecum gairhei* Junker, Brooks, and Boomker, 2008	Gharial, *Gavialis gangeticus* (Gmelin, 1789) (Crocodilia: Gavialidae)	Nepal	NHMUK 2008.6.16.1 (holotype); 2008.6.16.2-10 (paratypes)	Junker et al. (2008)
*Proctocaecum blairi* Tkach and Snyder, 2010	Freshwater crocodile	Northern Territory, Australia	QM G232133 (holotype); QM G232134, G232135, USNM 1397766, 1397767 (paratypes)	Tkach and Snyder (2010)
*Proctocaecum macroclemidis* (Tkach and Snyder, 2003) Brooks, 2004	Alligator snapping turtle, *Macrochelys temminckii* (Troost, 1835) (Testudines: Chelydridae)	Mississippi, US	USNM 1387303 (holotype); USNM 1387304, BMNH 2001.10.17.1-2 (paratypes)	Tkach and Snyder (2003)

a Type series destroyed during World War II (Brooks, 1980; p. 356).

b Additional paratypes in the collections of North Queensland Museum, and South Australian Museum.

Abbreviations: American Museum of Natural History, Division of Invertebrate Zoology, New York City, NY (AMNH); Natural History Museum, Parasitic Worms Collection, London, UK (NHMUK); Coleção Helmintológica do Instituto Oswaldo Cruz, Rio de Janeiro, Brazil (CHIOC); Meguro Parasitological Museum, Tokyo, Japan (MPM); Queensland Museum, Brisbane, Australia (QM); National Museum of Natural History’s Invertebrate Zoology Collection (USNM; Smithsonian Institution, Washington, DC).

Although >25 life cycles for fish-infecting cryptogonimids have been described, most of those are partial and/or experimental life cycles, did not emphasize morphological identification, or lack nucleotide information (Lundahl, 1941; Cable and Hunnien, 1942; Maillard, 1973, 1974; Cribb, 1986; Ostrowski de Núñez and Gil de Pertierra, 1991; Scholz et al., 1994; El-Darsh and Whitfield, 1999; Ostrowski de Núñez et al., 1999; Simões et al., 2008; Quintana and Ostrowski de Núñez, 2014; Quintana and Ostrowski de Núñez, 2016; Kvach et al., 2018; Jithila and Prasadan, 2022). To our knowledge, the study of Vélez-Sampedro et al. (2022) is the only complete life cycle of naturally infected (non-experimental) host using morphology and associated nucleotide information for a fish-infecting cryptogonimid.

In contrast to the fish-infecting cryptogonimids, current knowledge on the life histories of reptile-infecting cryptogonimids is scarce and incomplete. It comprises the experimental infection of 3 crocodilians (spectacled caiman, *Caiman crocodilus* Linnaeus, 1758 [Alligatoridae], Orinoco crocodile, *Crocodylus intermedius* Graves, 1819, and Morelet’s crocodile, *Crocodylus moreletii* [Duméril and Bibron, 1851] [Crocodylidae]) with metacercariae from freshwater fishes from Venezuela and Mexico (Ostrowski de Núñez, 1984; Salgado-Maldonado and Aguirre-Macedo, 1991), 1 snake (asiatic water snake, *Fowlea piscator* [Schneider, 1799] [Colubridae]) with metacercariae from a freshwater fish from India (Roopa and Janardanan, 1998), 1 hypothetical snake-infecting cercaria shed from the thorny tower snail, *Mieniplotia scabra* (Müller, 1774) (Gastropoda: Thiaridae) used to experimentally expose tadpoles of the Asian common toad, *Duttaphrynus melanostictus* (Schneider, 1799) (Anura: Bufonidae) from Sri Lanka (Jayawardena et al., 2013, 2017), and 2 hypothetically crocodilian-infecting metacercariae infecting fish from México (Martínez-Aquino et al., 2017). Ostrowski de Núñez (1987) successfully characterized for the first time a complete experimental life cycle of a reptile-infecting cryptogonimid, *Caimanicola brauni* (Mañé-Garzón and Gil, 1961) Brooks, 1980, by feeding a Hilaire’s side-necked turtle *Phrynops hilarii* (Dumeril and Birbon, 1835) (Chelidae) metacercariae recovered from a 10 spotted live-bearer, *Cnesterodon decemmaculatus* (Jenyns, 1842) (Actinopterygii: Poeciliidae), infected with cercariae from a cochliopid snail, *Heleobia castellanosae* (Gaillard, 1974) (Gastropoda: Cochliopidae) from a Buenos Aires Zoo (Argentina).

Our work herein is the first to characterize a complete naturally occurring life cycle for a reptile-infecting cryptogonimid, the first to be completed in North America, and the first to provide life history and nucleotide information for any species of *Proctocaecum*. In doing so, we provide a redescription of *P. diploporum*, supplemental description of *P. coronarium*, and discuss the taxonomy of *C. pavida* infecting the American alligator, based on morphology, *28S* nucleotide information and museum specimens.

## Materials and methods

### Specimen collection, preparation and deposition

Snails and fish were collected (7 Oct 2024, 12 Nov 2024 and 6 Mar 2025) from a saltmarsh pond that holds American alligators and that is intermittently connected to Bon Secour Bay (Mobile Bay, Alabama, north-central Gulf of America) during spring tides or storm events. Snails were collected from pond vegetation using kick nets, kept alive in pond water from the same pond they were collected, and transported to the Southeastern Cooperative Fish Parasite and Disease Laboratory (SCFPDL) at Auburn University (Auburn, AL, USA), where they were individually isolated in 6-well plates containing pond water from the same pond they were collected. The water was monitored daily under a stereo-dissecting microscope (Meiji RZ 3288, Meiji Techno Co., Saitama, Japan) to obtain naturally shed cercariae and subsequently the snails were cracked open for morphological identification and recovery of rediae. Fish were captured using standard minnow traps placed in shallow areas of the pond for 1–2 h, kept alive in pond water with aeration, and transported to the SCFPDL for identification and necropsy.

Fish were identified using morphological keys for the area (Boschung and Mayden, 2004); snails were identified based on shell and penis morphology following Heard et al. (2002).

Ten fish were whole fixed in 10% neutral-buffered formalin (n.b.f.) for histopathology. Fixed fish intended for histopathological examination were grossed to fit standard tissue processing cassettes, yielding 60 portions of tissue that were rinsed with distilled water for 2 h, immersed in Ethylenediaminetetraacetic acid (EDTA) for 3 weeks, processed routinely for histology by dehydration in an ethanol series, embedded in paraffin, sectioned at 4–5 μm, mounted on glass slides, dried and stained with hematoxylin and eosin (H&E) for tissue morphology (Luna, 1992; Gordillo-González et al., 2026).

Adult trematodes were collected opportunistically from American alligators on 2 occasions. The first collection occurred during an alligator hunt organized by the Alabama Department of Conservation and Natural Resources during 12–13 August 2022. Eight alligators were necropsied in the field at the weigh-in station in Spanish Fort (Mobile Bay area, Alabama, USA), where hunters delivered the alligators. The second collection occurred on 31 May 2023 where 3 fresh-dead adult alligators were collected and killed by personnel of the South Carolina Department of Conservation and Natural Resources at the Dennis Wildlife Center Fish Hatchery (Santee Cooper Reservoir, Cooper River drainage, South Carolina, USA). These specimens were transported to the SCFPDL for necropsy following SCFPDL standard necropsy procedures for intestinal parasite collection (Cajiao-Mora et al., 2024b).

Regarding larval stages collection, rediae and cercariae were observed alive in temporary mounts by pipetting them into a glass slide with pond water and a cover slip using an Olympus BX51 compound microscope (Olympus, Tokyo, Japan) equipped with differential interface contrast (DIC) optical components and a Jenoptik Gryphax camera (Jenoptik AG, Jena, Germany). Metacercariae were classified by size during necropsy as ‘large cyst’ (cyst diameter >240 μm) and ‘small cyst’ (<200 μm).

Specimens intended for morphological identification were heat-killed and fixed in 10% n.b.f. All other specimens were preserved in 95% non-denatured ethanol (EtOH) for DNA extraction. Fixed specimens were rinsed in water, stained overnight in Van Cleave’s and Ehrlich’s hematoxylins following Cajiao-Mora et al. (2024a), drawn using an Olympus BX51 compound microscope equipped with DIC and a drawing tube (Olympus U-DA Drawing Tube, Olympus, Tokyo, Japan), and selectively measured using a Jenoptik Gryphax camera. Measurements are reported in micrometres (μm), unless otherwise stated as the range followed by the mean in parentheses. Standard deviation is included when more than 10 characters are measured. Fixed specimens for scanning electron microscopy (SEM) were washed with de-ionized water, dehydrated through a graded EtOH series, pipetted onto a 45 μm mesh cut to fit within a 30 μm microporous specimen capsule, critical point dried in liquid CO_2_, mounted on SEM aluminium stubs with double-sided carbon tape, sputter-coated with gold palladium (19.32 g/cm^3^; 25 mA), and viewed with a Zeiss EVO 50VP SEM (Warren and Bullard, 2019). Nomenclature for Cryptogonimidae follows Miller and Cribb (2008b). Morphology follows Cajiao-Mora et al. (2025) and Truong et al. (2021; oesophagus length comprises the oesophagus from the posterior end of the oral sucker to the oesophageal bifurcation).

We examined the following museum specimens: vouchers of *P. coronarium* and *Timoniella loossi* (Viguera, 1957) Salgado-Maldonado and Aguirre-Macedo, 1991, and type series of *Caimanicola pavidus* (Brooks and Overstreet, 1977) Brooks, 1980 held in the National Museum of Natural History’s Invertebrate Zoology Collection (USNM, Smithsonian Institution, Washington, DC) and in the Harold W. Manter Laboratory of Parasitology (HWML, University of Nebraska, Lincoln, NE); vouchers of *Caimanicola marajoara* Teixeira de Freitas and Lent, 1938, type series of *Proctocaecum nicolli* Brooks, 1980, and *Proctocaecum macroclemidis* (Tkach and Snyder, 2003) Brooks, 2004, and paratypes of *Proctocaecum dorsale* Catto and Amato, 1993, and *Proctocaecum blairi* Tkach and Snyder, 2010 held in the USNM; type series of *P. diploporum* held in the American Museum of Natural History, Division of Invertebrate Zoology (AMNH, New York City, NY); microphotographs of the holotype of *C. marajoara* from the Helminthological Collection of the Oswaldo Cruz Institute (CHIOC, Rio de Janeiro, Brazil). Vouchers of each life stage obtained and described herein were deposited in the USNM; accession numbers are in Table 2.

**Table 2.** Museum and GenBank accession numbers of Cryptogonimidae species generated in this study Table 2 long description.

Species	Developmental stage	USNM museum accession numbers	*28S* GenBank accession numbers	*ITS2* GenBank accession numbers
*Proctocaecum diploporum* (Stunkard, 1931) Baugh, 1957	Redia	1774182–1774183	–	–
	Cercariae	1774176–1774181	PZ513850	PZ513906
	Metacercariae	1774172–1774175	PZ513907–PZ513912	PZ513851–PZ513856
	Adults	1774184–1774186	PZ513849	PZ513905
*Proctocaecum coronarium* (Cobbold, 1861) Brooks, 1980	Redia	1774211	PZ513180	PZ513218
	Cercariae	1774206–1774210	PZ513172	PZ513210
	Metacercariae	1774200–1774205	PZ513173–PZ513179	PZ513211–PZ513217
	Adults	neotype: 1774187; vouchers: 1774188–1774199	PZ513171	PZ513209
*Proctocaecum macroclemidis* (Tkach and Snyder, 2003) Brooks, 2004	Adults	1774212–1774214	PZ507502	PZ507503

Abbreviation: National Museum of Natural History’s Invertebrate Zoology Collection (USNM; Smithsonian Institution, Washington, DC).

### DNA extraction

A total of 3 rediae, 4 cercariae, 12 small cyst metacercariae, 12 large cyst metacercariae, and 5 adults preserved in EtOH were used for DNA extraction and sequencing. Extraction was performed using the DNeasy^TM^ Blood and Tissue kit (Qiagen) following the manufacturer’s protocol, except that the proteinase-K incubation period was extended overnight. Once extracted, DNA concentration was measured using a NanoDrop-One Microvolume Spectrophotometer (Thermo Fisher Scientific), diluted to 50 ng/μL, and stored at −20°C. The partial *28S* gene was amplified using the forward primer U178 (5’-GCACCCGCTGAAYTTAAG-3’), and reverse primer L1642 (5’-CCAGCGCCATCCATTTTCA-3’), sequencing primers 300 F (5’-CAAGTACCGTGAGGGAAAGTTG-3’) and 1200 R (5’-GCATAGTTCACCATCTTTCGG-3’) (Lockyer et al., 2003). The *ITS2* region was amplified using forward primer GA1 (5’-AGAACATCGACATCTTGAAC-3’; Anderson and Barker, 1998) and reverse primer ITS2.2 (5’-CCTGGTTAGTTTCTTTTCCTCCGC-3’; Cribb et al., 1998). Polymerase chain reaction (PCR) reactions were performed following Truong et al. (2021). DNA amplification was verified with a 1% agarose gel stained with ethidium bromide. PCR products were purified using the QIAquick PCR Purification Kit (Qiagen) according to the manufacturer’s protocol, except that the last elution step was performed with autoclaved nanopure water. DNA sequencing was performed by Genewiz (South Plainfield, New Jersey). Sequence assembly and analysis of chromatograms were performed with Geneious prime version 2023.2.1. Forward and reverse sequences were aligned using MAFFT tool (Katoh and Standley, 2013) and low-quality read-ends were trimmed. All sequences were deposited in GenBank; accession numbers are in Table 2.

### Phylogenetic analysis

We performed maximum likelihood tree inference using *28S* sequences. The *ITS2* phylogeny did not show useful resolution and therefore is not shown herein. The ingroup taxa of the *28S* analysis comprised our newly generated sequences, including a sequence of a specimen of *Proctocaecum macroclemidis* (Tkach and Snyder, 2003) Brooks, 2004 that the authors collected from an alligator snapping turtle (*Macrochelys temminckii* Troost,1835 [Chelydridae]) from Alabama (the identification was based on comparisons with the holotype USNM 1387303; see description in Tkach and Snyder, 2003), and those ascribed to Cryptogonimidae following Kmentová et al. (2020), Yong et al. (2023) and Cajiao-Mora et al. (2025). We excluded sequences shorter than 1000 base pairs (bp). The outgroup follows Yong et al. (2023). Sequences were aligned using the multiple alignment using fast Fourier transform (MAFFT) tool (Katoh and Standley, 2013), and the alignment was trimmed to our shortest sequence (1145 bp). The phylogeny was inferred using IQTREE v.1.16.12 (Nguyen et al., 2015). Substitution model testing was done with ModelFinder (Kalyaanamoorthy et al., 2017) as implemented in IQTREE. After model testing, tree inference was done using best-fitting substitution models (Chernomor et al., 2016). Default tree search parameters were used, except perturbation strength was set to 0.2 and 500 iterations had to be unsuccessful to stop the tree search. Tree inference was done 20 times with only the tree with the best log-likelihood score reported. Support for relationships was measured with 1000 ultrafast bootstrap replicates (UFBoot) (Hoang et al., 2018). The inferred phylogenetic tree was visualized using FigTree v1.4.4 (Rambaut et al., 2018) and further edited for visualization purposes with Adobe Illustrator (Adobe Systems).

## Results

### *Proctocaecum coronarium (Cobbold, 1861) Brooks, 1980 (Cryptogonimidae)* (Figures 1, 2, 3A, 4A and 4C)

#### Redia (Figure 1A)

*Host: Littoridinops monroensis* (Frauenfeld, 1863) (Gastropoda: Cochliopidae).

**Figure 1.**
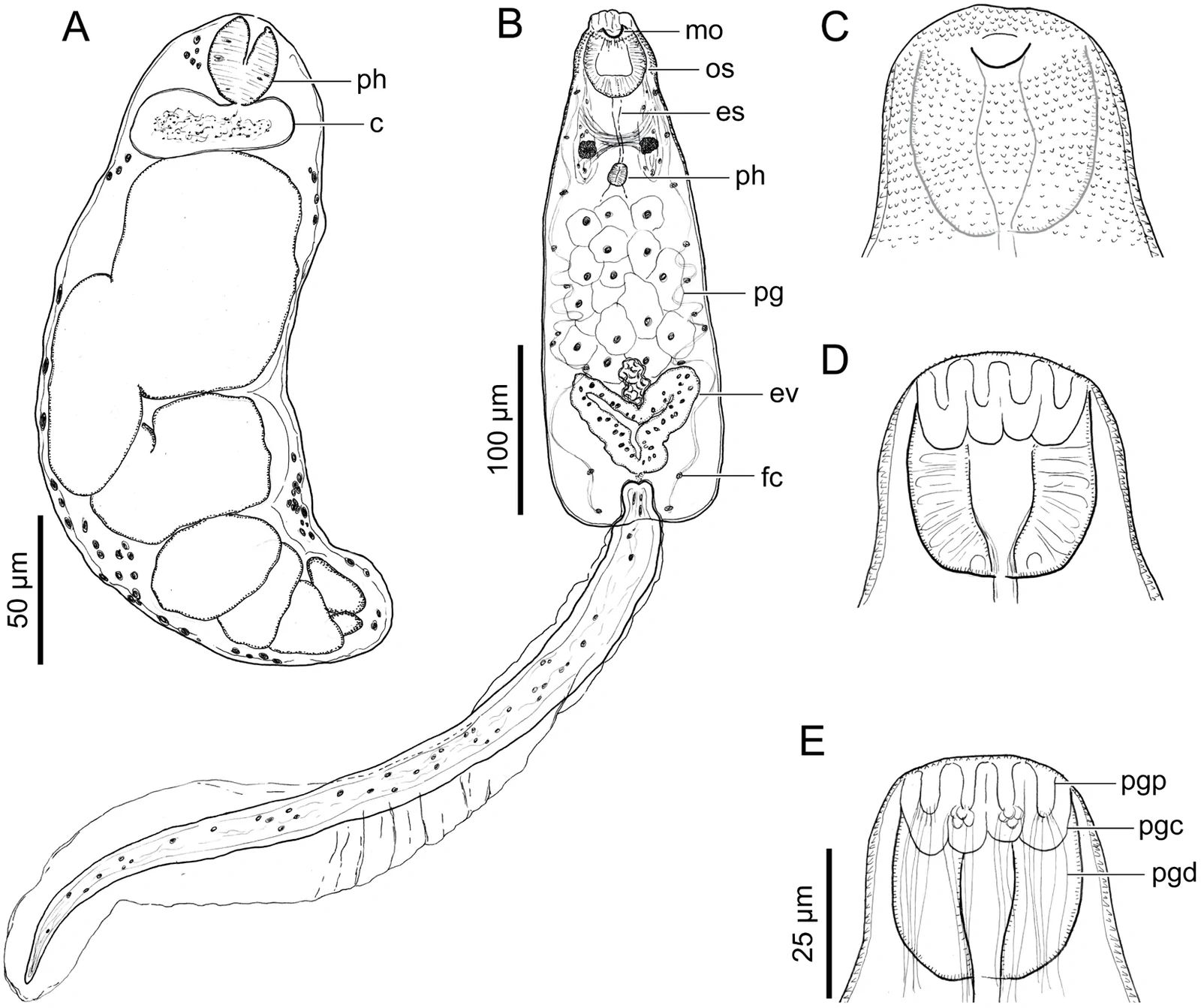
*Proctocaecum coronarium* (Cobbold, 1861) Brooks, 1980 (Digenea: Cryptogonimidae) from *Littoridinops monroensis* (Frauenfeld, 1863) (Gastropoda: Cochliopidae) from a saltmarsh pond in the Bon Secour Bay area, Alabama, USA. Scale value aside each bar. (A) Redia, ventral view (voucher USNM 1774211). (B) Cercaria, ventral view (voucher USNM 1774210). (C) Cercaria, anterior end, ventral view (voucher USNM 1774207). (D) Cercaria, anterior end, medial view (voucher USNM 1774207). (E) Cercaria, anterior end, dorsal view (voucher USNM 1774207). Abbreviations: cecum (c); oesophagus (es); excretory vesicle (ev); flame cells (fc); mouth (mo); oral sucker (os); penetration glands (pg); penetration gland channel (pgc); penetration gland duct (pgd); penetration gland pore (pgp); pharynx (ph).

*Locality*: Bon Secour Bay, Alabama (USA).

*Site in host/tissue infected*: Digestive gland.

*Prevalence and intensity*: 3 of 300 (1%) snails were infected with 2–5 rediae each.

*Specimens deposited*: USNM 1774211.

*Representative DNA sequences: 28S* GenBank accession No. PZ513180, *ITS2* GenBank accession No. PZ513218.

*Description (based on 1 heat-killed, stained and permanently whole-mounted redia plus 3 live, wet-mounted rediae)*: Body ovoid in outline, 253–456 (3) in total length, 83–164 (3) in maximum width. Oral opening terminal. Pharynx ovoid, 27 (1) long, 22 (1) wide. Cecum sac-like, immediately post-pharyngeal, transversally elongated. Birth pore posterior to pharynx, lateral to cecum. Body containing several developing cercariae; larger cercariae in anterior portion (Figure 1A).

#### Cercaria (Figures 1B–E)

*Host*: Littoridinops monroensis (Frauenfeld, 1863) (Gastropoda: Cochliopidae).

*Locality*: Bon Secour Bay, Alabama (USA).

*Site in host/tissue infected*: Digestive gland.

*Prevalence and intensity*: 3 of 300 (1%) snails shed 5–10 cercariae each in 7 days; each snail was infected with 10–20 cercariae upon being necropsied.

*Specimens deposited*: USNM 1774206–1774210.

*Representative DNA sequences: 28S* GenBank accession No. PZ513172, *ITS2* GenBank accession No. PZ513210.

*Description (based on 5 heat-killed, stained and permanently whole-mounted cercariae plus 10 live, wet-mounted cercariae)*: Pleurolophocercous. Body ovoid in outline, 775–819 (794; 4) in total length, 96–112 (106; 5) in maximum width, 7 × longer than wide. Tegument spinose, containing few hair-like sensory structures. Tegumental spines visible in anterior end of body, becoming smaller posteriad, extending close to eyespots level (Figure 1C). Hair-like sensory structures 25–30 (1) in total length, distributing along ventral lateral body margin, herein indistinct except in live cercariae observed with DIC. Tail 472–554 (502; 4) in total length, 32–40 (36; 4) in maximum width, having dorsoventral fin-folds (Figure 1B); fin-folds extending from anterior quarter of tail to end of tail, becoming confluent at end of tail (Figure 1B). Primordial ventral sucker located immediately anterior to excretory vesicle (Figure 1B). Oral sucker subterminal, ovoid in outline (Figures 1B–E), 37–47 (44; 5) in total length, 34–37 (35; 5) in maximum width. Oesophagus narrow (Figure 1B). Pharynx ovoid in outline (Figure 1B). Ceca not observed. Eye spots 2, in anterior portion of body, flanking oesophagus, between pharynx and oral sucker (Figure 1B). Penetration glands occupying middle third of body (Figure 1B), at least 7 pairs observed, ovoid to irregular in outline, having spheroid nucleus (Figure 1B), with ducts extending anteriad; penetration gland ducts dorsal to oral sucker, becoming confluent at level of oral sucker, forming 4 channels (Figures 1D and 1E); channels having a terminal pore (Figures 1D and 1E). At least 10 flame cells per side of body observed in live cercariae, distributing along lateral body margin (Figure 1B). Excretory vesicle Y-shaped, in posterior portion of body, posterior to penetration glands, (Figure 1B); excretory vesicle wall 11–14 (12; 3) thick. Cercarial behaviour comprising rapid, irregular tail movements in no particular vertical direction for 1–3 sec then a pause in activity and followed by cercarial sinking for 3–5 sec before restarting cycle.

#### Metacercaria (Figures 2A and 3A)

*Hosts*: Sheepshead minnow, *Cyprinodon variegatus* Lacepède, 1803 (Cyprinodontiformes: Cyprinodontidae); sailfin molly, *Poecilia latipinna* (Lesueur, 1821) (Cyprinodontiformes: Poeciliidae); rainwater killifish, *Lucania parva* (Baird and Girard, 1855) (Cyprinodontiformes: Fundulidae); gulf killifish, *Fundulus grandis* Baird and Girard, 1853 (Cyprinodontiformes: Fundulidae); bayou killifish, *Fundulus pulvereus* (Evermann, 1892); inland silverside, *Menidia beryllina* (Cope, 1867) (Atheriniformes: Atherinopsidae); striped mullet, *Mugil cephalus* Linnaeus, 1758 (Mugiliformes: Mugilidae).

**Figure 2.**
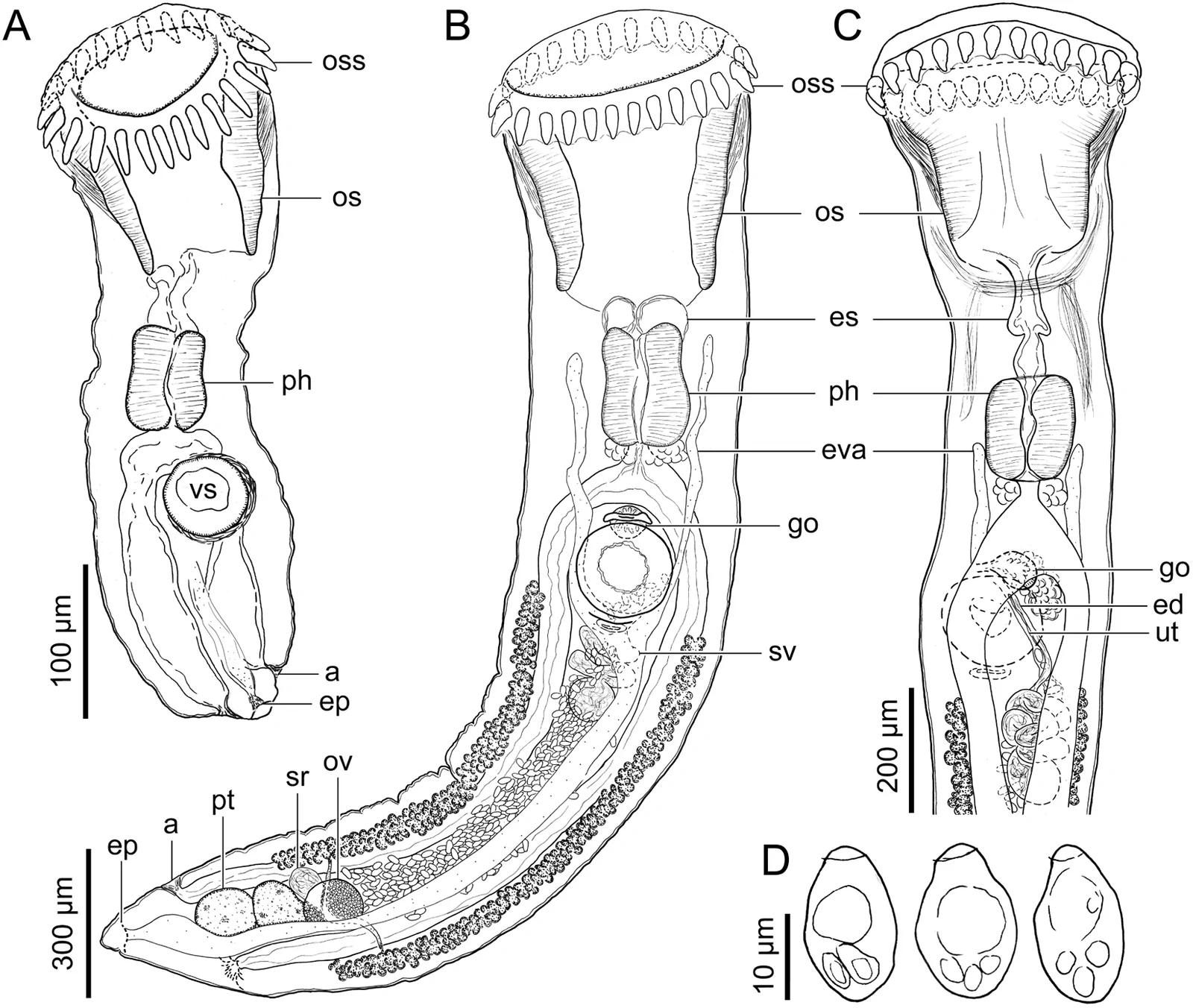
*Proctocaecum coronarium* (Cobbold, 1861) Brooks, 1980 (Digenea: Cryptogonimidae). Scale value aside each bar. (A) Metacercaria (voucher USNM 1774202) excysted from within a scale of sheepshead minnow, *Cyprinodon variegatus* Lacepède, 1803 (Cyprinodontiformes: Cyprinodontidae) from a saltmarsh pond in the Bon Secour Bay area, Alabama, USA. Ventral view. (B) Adult (neotype USNM 1774187) infecting American alligator, *Alligator mississippiensis* (Daudin, 1802) (Crocodilia: Alligatoridae) from Alabama, USA. Ventral view. (C) Adult (voucher USNM 1774188) infecting American alligator, from Alabama, USA. Anterior end, dorsal view. (D) Eggs (voucher USNM 1774193). Abbreviations: anal pore (a); oesophagus (es); ejaculatory duct (ed); excretory pore (ep); excretory vesicle arms (eva); gonotyl (go); oral sucker (os); oral sucker spines (Oss); ovary (ov); pharynx (ph); posterior testis (pt); seminal receptacle (sr); seminal vesicle (sv); uterus (ut); ventral sucker (vs).

**Figure 3.**
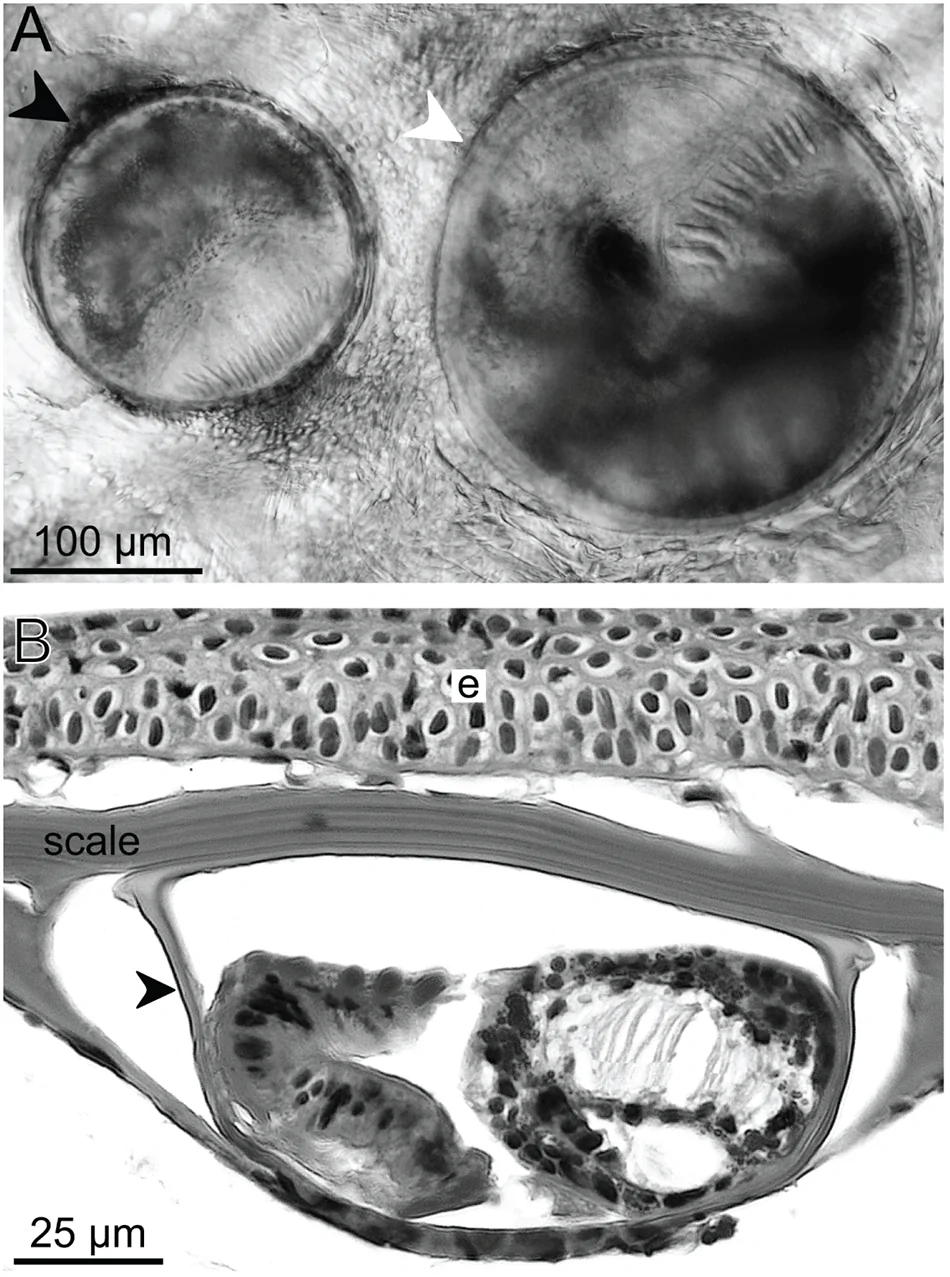
Encysted metacercariae. Scale value aside each bar. (A) Encysted metacercariae of *Proctocaecum diploporum* (Stunkard, 1931) Baugh, 1957 (Digenea: Cryptogonimidae) (black arrowhead) and *Proctocaecum coronarium* (Cobbold, 1861) Brooks, 1980 (white arrowhead) within the scale of a gulf killifish, *Fundulus grandis* Baird and Girard, 1853 (Cyprinodontiformes: Fundulidae) from a saltmarsh pond in the Bon Secour Bay area, Alabama, USA. (B) Histological section stained with hematoxylin and eosin of a scale of a sheepshead minnow, *Cyprinodon variegatus* Lacepède, 1803 (Cyprinodontiformes: Cyprinodontidae) from a saltmarsh pond in the Bon Secour Bay, with an encysted metacercaria of *Proctocaecum* sp. (black arrowhead). Abbreviations: epithelium (e).

*Locality*: Bon Secour Bay, Alabama (USA).

*Site in host*: Within scales, in the fins enveloped by lepidotrichia, and in the epidermis between fin rays.

*Prevalence and intensity*: We examined ∼5–20 specimens of each fish species; each individual of each fish species was infected (100% prevalence) with approximately 5–50 cysts.

*Specimens deposited*: USNM 1774200–1774205.

*Representative DNA sequences: 28S* GenBank accession No. PZ513173–PZ513179, *ITS2* GenBank accession No. PZ513211–PZ513217.

*Description (based on 22 encysted metacercariae, 4 excysted, heat-killed, stained and permanently whole-mounted metacercariae, and 10 excysted, live, wet-mounted metacercariae)*: Cyst spheroid, membranous-like, transparent (Figure 3A), 244–292 (265 ± 14; 22) by 232–292 (261 ± 15; 22), within fish scales and fin rays. Excysted metacercaria 420–471 (449; 4) in total length, 140–166 (156; 4) in maximum width. Tegument spinose; tegumental spines visible in anterior third of body. Ventral sucker spheroid, equatorial to slightly post-equatorial (Figure 2A), 49–65 (60; 4) in total length, 54–66 (60; 4) in maximum width. Oral sucker terminal, infundibular, 117–135 (127; 4) in total length, 140–166 (156; 4) in maximum width, having circumoral spines (Figure 2A); oral sucker spines 24 in number, widest at insertion, tapering posteriad (Figure 2A), 27–31 (29; 31) in total length, 6–8 (7; 31) width at insertion. Oesophagus medial (Figure 2A). Pharynx quadrate in outline, having blunt corners, 67–77 (74; 4) in total length, 35–50 (44; 4) in maximum width (Figure 2A). Intestine bifurcating after pharynx (Figure 2A). Ceca extending posteriad in parallel with lateral body margins; each cecum opening through a posterolateral pore (anal pore). Excretory vesicle Y-shaped, extending from posterior body end to posterior end of oral sucker, bifurcating posterior to ventral sucker; excretory pore sub-terminal, dorsal (Figure 2A).

#### Adult (Figures 2B–D, 4A and 4C; Table 3)

*Type host and host reported herein*: American alligator, *Alligator mississippiensis* (Daudin, 1802) (Crocodilia: Alligatoridae).

**Table 3.** Comparative measurements of adult Cryptogonimidae: *Proctocaecum coronarium* (Cobbold, 1861) Brooks, 1980, *Proctocaecum diploporum* (Stunkard, 1931) Baugh, 1957, and *P. diploporum* (as *Caimanicola pavida* [Brooks and Overstreet, 1977] Brooks, 1980) infecting American alligator, *Alligator mississippiensis* (Daudin, 1802) (Crocodilia: Alligatoridae) Table 3 long description.

Species	*P. coronarium*	*P. coronarium*	*P. diploporum*	*P. diploporum*	*P. diploporum as C. pavida*
**Host**	American alligator	American alligator	American alligator	American alligator	American alligator
**Locality**	Alabama and South Carolina, USA	Menagerie of the Zoological Society of London (London Zoo), London, UK	Alabama and South Carolina, USA	Unknown	Louisiana, Mississippi, and Florida, USA
**Source**	Present study	Cobbold (1861)	Present study	Stunkard (1931)	Brooks and Overstreet (1977)
*N*	8	‘Numerous’	3	5	30
Total body length	1.7– 2.5 (2.3; 8) mm	6.35 mm	4.0–6.4 (5.5; 3) mm	2.6–3.3 mm	1.6–10.6 mm
Forebody length	421– 986 (753; 8)		1379–2589 (2158; 3)		24–49% (35%) of TBL
Hindbody length	957–1578 (1302; 8)		2510–3654 (3143; 3)		
Body maximum width	340–490 (440; 7) at OS	725	418–538 (483; 3) at VS	300–390	400–1100
Width at intestinal bifurcation	236–424 (340; 7)		374–518 (447; 3)		
Width at ovary level	184–357 (295; 7)		353–467 (424; 3)		
Width at anal pores	119–245 (198; 7)		108–153 (134; 3)		
Dextral anal pore to PEB	114–266 (176; 7)		Terminal		
Sinistral anal pore to PEB	124–283 (213; 7)		Terminal		
Oral sucker length	343–517 (449; 8)		396–473 (439; 3)	250–320	195–575
Oral sucker max width	340–490 (437; 8)		340–391 (370; 3)	140–210	230–552
Number circumoral spines	24 (8)	24	26–27 (2)	24	26–28
Ventral circumoral spines length	43–73 (62; 7)		56–61 (59; 8)	44–58	45–77
Ventral circumoral spines width at insertion	15–32 (22; 7)		18–19 (19; 8)		12–27
Dorsal circumoral spines length	47 (1)		–		53–85
Dorsal circumoral spines width at insertion	26 (1)		–		12–27
Ventral sucker length	126–193 (157; 8)		168–216 (194; 3)	130–180	138–310
Ventral sucker width	125–198 (160; 7)		166–218 (191; 3)		72–322
Oesophagus total length	411–446 (429; 2)		863–2012 (1628; 3)		
Pre-pharyngeal oesophagus length	203–260 (232; 2)		147–446 (281; 3)		120–402
Post-pharyngeal oesophagus length	29–50 (37; 5)		543–1517 (1141; 3)		180–689
Pharynx length	155–232 (180; 8)		171–219 (202; 189)		126–345
Pharynx width	128–198 (160; 8)		176–206 (189; 3)	130–146	115–345
Gonotyl length	43–99 (64; 8)		–		
Gonotyl width	52–92 (70; 6)		–		
Gonotyl depth	68 (1)		–		
Anterior testis length	76–133 (102; 8)		158–253 (221; 3)		149–460
Anterior testis width	79–143 (113; 8)		155–212 (192; 3)	140–210	161–568
Posterior testis length	88–151 (114; 8)		180–303 (257; 3)		148–575
Posterior testis width	87–124 (108; 8)		160–217 (192; 3)		172–483
Ovary length	83–120 (103; 8)		201–233 (215; 3)		115–379
Ovary width	62–105 (91; 8)		122–171 (154; 3)	110–180	149–425
Ovary to AEB	1353–2085 (1805; 8)		3316–5475 (4660; 3)		
Ovary to PEB	286–503 (388; 8)		521–737 (643; 3)		
Oviducal seminal receptacle length	81––134 (104; 7)		86–105 (96; 2)		86–316
Oviducal seminal receptacle width	58–98 (74; 7)		51–92 (72; 2)	70–85	53–237
Vitellarium length	673–1203 (990; 8)		1750–2437 (2205; 3)		
Anterior end to vitellarium length	780–1133 (944; 8)		2026–3466 (2946; 3)		
Posterior end to vitellarium length	217 –464 (330; 8)		331–420 (370; 3)		
Egg length	23–26 (24; 29)		23–27 (25; 13)	20–28	32–35
Egg width	10–14 (12; 29)		12–16 (14; 13)	11–13	15–17
Tegumental spines length before pharynx	1–3 (2; 5)		10 (1)		
Tegumental spines length at pharynx	2–4 (3; 4)		14 (1)		
Tegument spines length at VS	2–5 (4; 4)		7 (1)		5–19

Abbreviations: anterior end of body (AEB); oral sucker (OS); posterior end of body (PEB); ventral sucker (VS).

*Type locality*: Alabama (USA)

*Localities reported herein*: Alabama (USA); Dennis Wildlife Center Fish Hatchery (Santee Cooper Reservoir, Cooper River drainage, South Carolina, USA)

*Site in host*: Intestine.

*Prevalence and intensity*: 3 of 11 (27%) alligators were infected with 5–10 *P. coronarium*

*Specimens examined*: vouchers of *P. coronarium* USNM 1381392, 1385103 1356162; HWML 20854

*Specimens deposited*: Neotype USNM 1774187; vouchers USNM 1774188–1774199.

*Representative DNA sequences: 28S* GenBank accession No. PZ513171, *ITS2* GenBank accession No. PZ513209.

**Figure 4.**
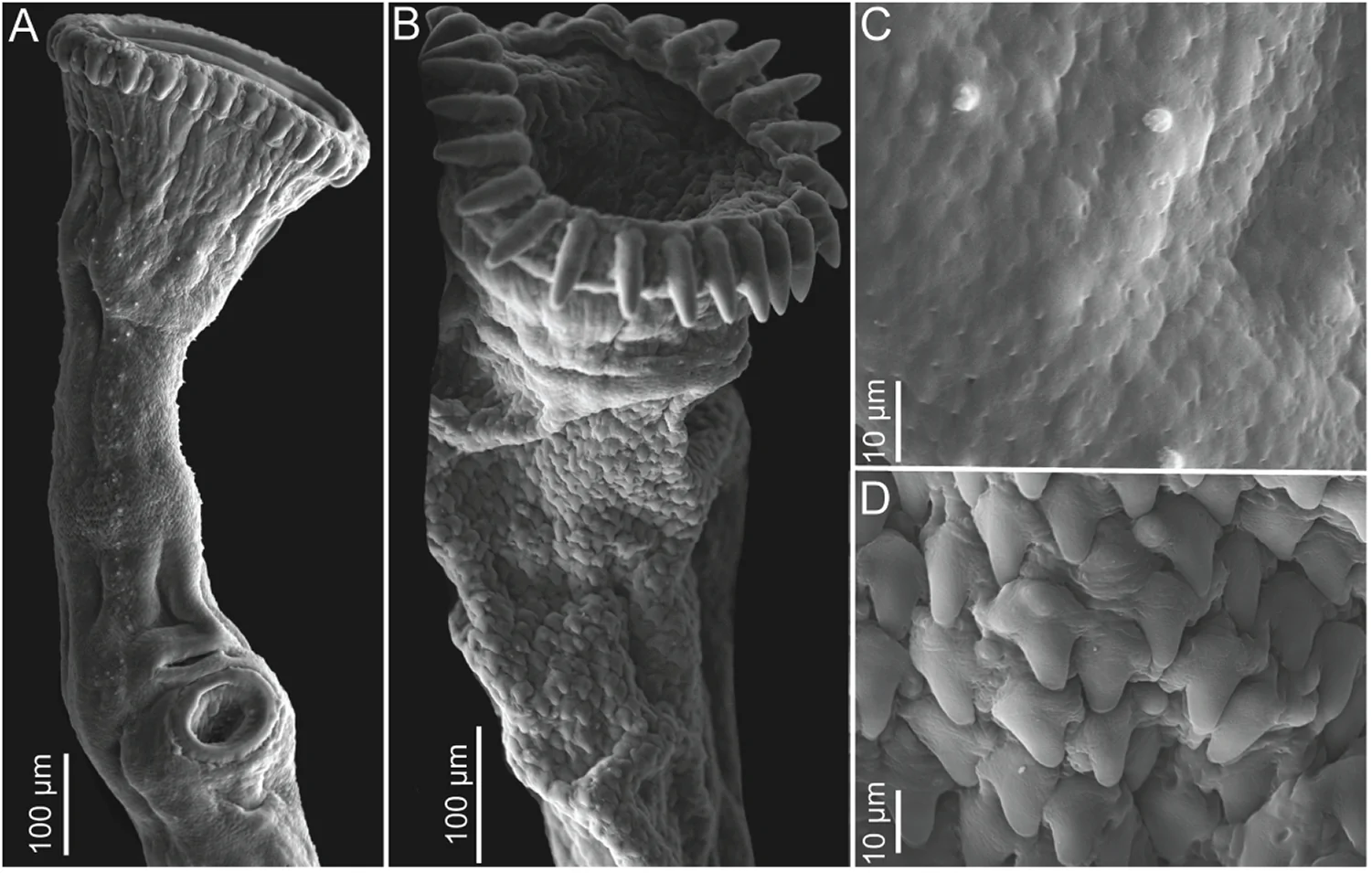
Scanning electron microscopy (SEM) of adult *Proctocaecum coronarium* (Cobbold, 1861) Brooks, 1980 (Digenea: Cryptogonimidae) and *Proctocaecum diploporum* (Stunkard, 1931) Baugh, 1957 from American alligators, *Alligator mississippiensis* (Daudin, 1802) (Crocodilia: Alligatoridae) from South Carolina and Alabama, USA. Scale value aside each bar. (A) Anterior end of *P. coronarium*. (B) Anterior end of *P. diploporum*. (C) Body surface spines from anterior end of body of *P. coronarium*. (D) Body surface spines from anterior end of body of *P. diploporum*.

*Supplemental description (based on 8 heat-killed, stained and permanently whole-mounted adults)*: Body elongate, having a pointed posterior end (Fig. 2B), 5–6× longer than wide. Forebody 20–38% (32%; 8) of total body length. Hindbody 52–63% (56%; 8) of total body length. Tegument spinose, having 2 tegumental infoldings (Figures 4A and 4C); body surface spines minute (Figure 4C), scale-like, becoming larger at level of ventral sucker, then becoming smaller and fewer posteriad, extending to near posterior end of body; tegumental infoldings anterior and posterior to ventral sucker (Figure 4A). Ventral sucker spherical, in first quarter of body (Figure 2B). Oral sucker terminal, infundibular, having circumoral spines (Figures 2B, 2C, 4A); oral sucker spines widest at insertion, tapering posteriad (Figures 2B, 2C and 4A). Oesophagus medial, representing 17–19% (18%; 2) of total body length, comprising pre-pharyngeal, pharyngeal and post-pharyngeal parts (Figures 2B and 2C). Pre-pharyngeal oesophagus retractile, 49–58% (54%; 2) of total oesophagus length. Pharynx quadrate in outline, having blunt corners (Figures 2B and 2C), surrounding oesophagus, representing 38–41% (39%; 2) of total oesophagus length. Post-pharyngeal oesophagus very short, surrounded by clusters of glandular-like cells (Figures 2B and 2C), 9–10% (9%; 2) of total oesophagus length. Intestine bifurcating shortly after pharynx (Figures 2B and 2C), 30–36% (33%; 8) of body length to anterior body end. Ceca slender, extending posteriad parallel to lateral body margins, asymmetrical in length, symmetrical in width, each cecum opening through posterolateral pore (anal pore); anal pores asymmetrical in position, 1 pore at level of posterior testis, other pore posterior to posterior testis (Figures 2B).

Testes 2, spherical, medial, in last quarter of body, in tandem, contiguous with ovary (Figure 2B). Vasa efferentia obscured by dense mass of eggs through its length. Seminal vesicle long, sinuous, extending posterior to ventral sucker (Figures 2B and 2C); having convoluted proximal portion (Figure 2B and 2C), having distal portion enlarged, sac-like (Figures 2B and 2C); prostatic duct not observed; ejaculatory duct long, opening into genital pore through a hermaphroditic duct (Figure 2C). Genital pore medial, in anterior tegumental infolding, between ventral sucker and gonotyl (Figure 2C). Gonotyl spheroid, muscular, protractile, surrounded by clusters of glandular-like cells (Figures 2B and 2C)

Ovary spheroid (Figure 2B); pre-ovarian space 76–83% (78%; 8) of total body length; post-ovarian space 13–20% (17%; 8) of total body length. Oviduct emerging dorsally from ovary. Oviducal seminal receptacle sac-like, postero-dorsal to ovary. Laurer’s canal, oötype, and Mehlis’ gland not observed. Uterus coiling anteriorly between ceca, extending from ovary to common genital pore, having a convoluted proximal portion, having a sinuous to straight distal portion (Figures 2B and 2C), ventral to seminal vesicle, with numerous eggs (Figure 2B), confluent with hermaphroditic duct. Hermaphroditic duct opening into genital pore (Figure 2C). Eggs ovoid, operculate (Figure 2D). Vitellarium follicular, distributing in 2 bilaterally symmetrical fields, extending from anterior margin of anterior testis to posterior margin of ventral sucker (Figure 2B), occupying 36–50% (42%; 8) of total body length; pre-vitellarium space 34–50% (41%; 8) of total body length; post-vitellarium space 10–18% (14%; 8) of total body length. Transverse vitelline ducts emanating from posterior quarter of vitellarium, at level of ovary, forming vitelline reservoir; vitelline reservoir dorsal to ovary, connecting with oviduct (Figure 2B).

Excretory vesicle Y-shaped, ventral, extending from posterior body end to pharynx (Figures 2B and 2C), bifurcating posterior to ventral sucker (Figure 2B); excretory pore sub-terminal, dorsal (Figure 2B).

### *Proctocaecum diploporum (Stunkard, 1931) Baugh, 1957 (Cryptogonimidae)* (Figures 3A, 4B, 4D, 5 and 6A–B)

#### Redia (Figure 5A)

*Host: Littoridinops monroensis* (Frauenfeld, 1863) (Gastropoda: Cochliopidae).

*Locality*: Bon Secour Bay, Alabama (USA).

*Site in host/tissue infected*: Digestive gland.

*Prevalence and intensity*: 1 of 300 (0.3%) snails was infected.

*Specimens deposited*: USNM 1774182, 1774183.

**Figure 5.**
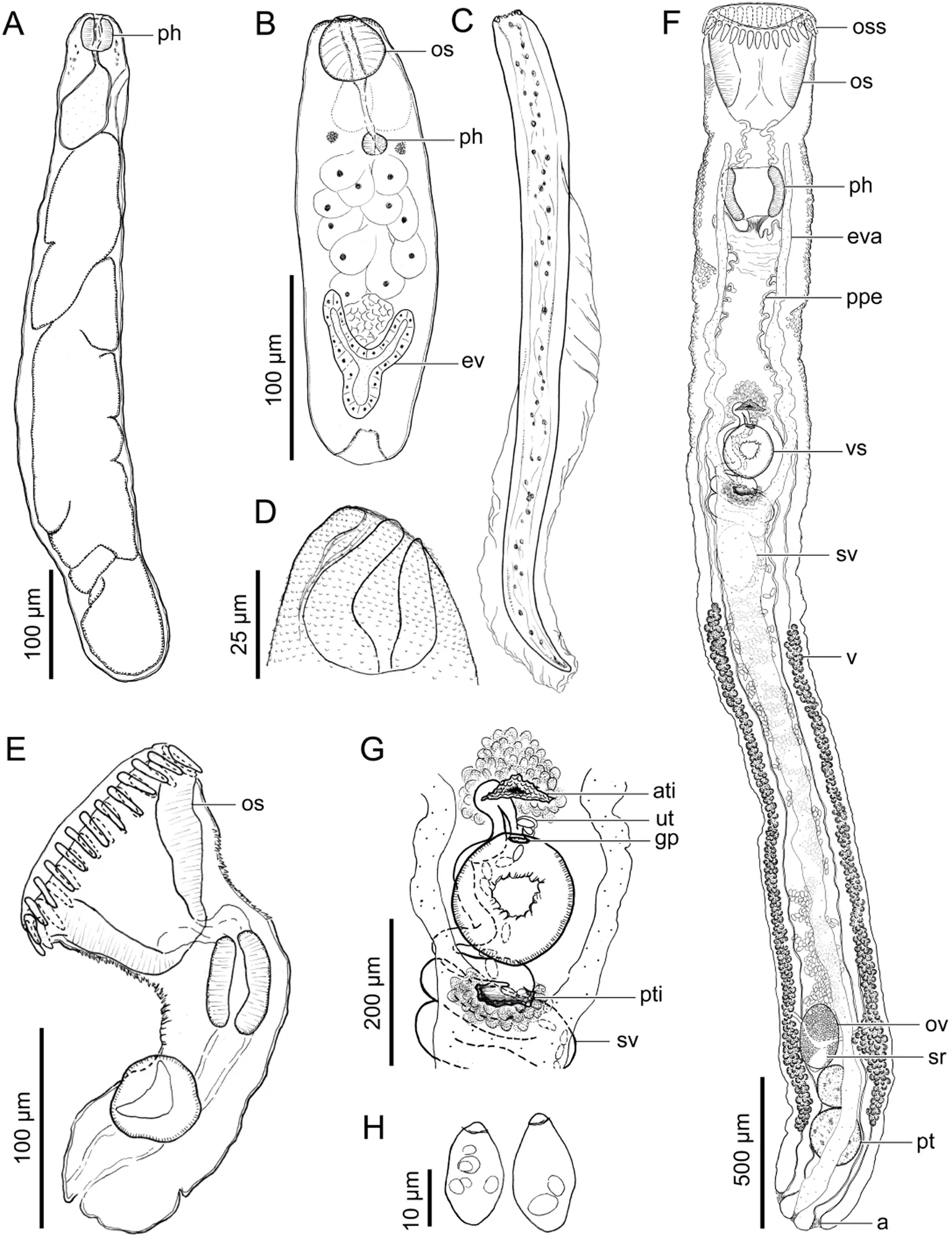
*Proctocaecum diploporum* (Stunkard, 1931) Baugh, 1957 (Digenea: Cryptogonimidae). Scale value aside each bar. (A) Redia (voucher USNM 1774183) from *Littoridinops monroensis* (Frauenfeld, 1863) (Gastropoda: Cochliopidae) from a saltmarsh pond in the Bon Secour Bay area, Alabama, USA. Ventral view. (B) Cercarial body (voucher USNM 1774180) from *L. Monroensis*. Ventral view. (C) Cercarial tail (voucher USNM 1774179). (D) Anterior end of cercaria (voucher USNM 1774179). (E) Metacercaria (voucher USNM 1774175) excysted from within a scale of sheepshead minnow, *Cyprinodon variegatus* Lacepède, 1803 (Cyprinodontiformes: Cyprinodontidae) from a saltmarsh pond in the Bon Secour Bay area, Alabama, USA. Lateral view. (F) Adult (voucher USNM 1774186) infecting American alligator, *Alligator mississippiensis* (Daudin, 1802) (Crocodilia: Alligatoridae) from Alabama, USA. Ventral view. (G) Close view of male and female terminal genitalia (voucher USNM 1774186). Ventral view. (H) Eggs (voucher USNM 1774186). Abbreviations: anal pore (a); anterior tegumental infolding (ati); excretory vesicle (ev); excretory vesicle arms (eva); genital pore (gp); oral sucker (os); oral sucker spines (Oss); ovary (ov); pharynx (ph); post-pharyngeal oesophagus (ppe); posterior tegumental infolding (pti); posterior testis (pt); seminal receptacle (sr); seminal vesicle (sv); uterus (ut); ventral sucker (vs); vitellarium (v).

*Description (based on 1 heat-killed, stained and permanently whole-mounted redia)*: Body elongate, 619 (1) in total length, 106 (3) in maximum width. Oral opening terminal. Pharynx ovoid, 30 (1) long, 31 (1) wide. Cecum sac-like, immediately post-pharyngeal. Birth pore lateral, at level of cecum. Body containing several developing cercariae; larger cercariae in anterior portion (Figure 5A).

#### Cercaria (Figures 5B–D)

*Host: Littoridinops monroensis* (Frauenfeld, 1863) (Gastropoda: Cochliopidae).

*Locality*: Bon Secour Bay, Alabama (USA).

*Site in host/tissue infected*: Digestive gland.

*Prevalence and intensity*: 1 of 300 (0.3%) snails was infected with ∼11 cercariae.

*Specimens deposited*: USNM 1774176–1774181.

*Representative DNA sequences: 28S* GenBank accession No. PZ513850, *ITS2* GenBank accession No. PZ513906.

*Description (based on 3 heat-killed, stained and permanently whole-mounted cercariae*): Pleurolophocercous. Body ovoid in outline (Figure 5B), 226–250 (239; 3) length without the tail, 63–70 (67; 4) in maximum width. Tegument spinose; tegumental spines scale like, visible in anterior end of body, becoming smaller and fewer posteriad, extending close to eyespots level (Figure 5D). Tail 360–364 (362; 2) in total length, 28–31 (30; 2) in maximum width, having dorsoventral fin-folds (Figure 5C); fin-folds extending from anterior quarter of tail to end of tail, becoming confluent at end of tail (Figure 5C). Primordial ventral sucker located immediately anterior to excretory vesicle (Figure 5B). Oral sucker subterminal, spheroid (Figures 5B and 5D), 34–36 (35; 3) in total length, 29–36 (33; 3) in maximum width. Oesophagus narrow (Figure 5B). Pharynx small (Figure 5B). Ceca not observed. Eye spots 2, in anterior portion of body, flanking oesophagus. Penetration glands occupying middle third of body (Figure 5B), ovoid to irregular in outline, having spheroid nucleus (Figure 5B), with ducts extending anteriad; penetration glands ducts dorsal to oral sucker, becoming confluent at level of oral sucker; ducts having a terminal pore (Figure 5D). Flame cells not observed. Excretory vesicle Y-shaped, in posterior portion of body, posterior to penetration glands, thick walled (Figure 5B). Cercarial behaviour not observed.

#### Metacercaria (Figures 3A and 5E)

*Hosts*: Sheepshead minnow, *Cyprinodon variegatus* Lacepède, 1803 (Cyprinodontiformes: Cyprinodontidae); sailfin molly, *Poecilia latipinna* (Lesueur, 1821) (Cyprinodontiformes: Poeciliidae); rainwater killifish, *Lucania parva* (Baird and Girard, 1855) (Cyprinodontiformes: Fundulidae); gulf killifish, *Fundulus grandis* Baird and Girard, 1853 (Cyprinodontiformes: Fundulidae); bayou killifish, *Fundulus pulvereus* (Evermann, 1892); inland silverside, *Menidia beryllina* (Cope, 1867) (Atheriniformes: Atherinopsidae); striped mullet, *Mugil cephalus* Linnaeus, 1758 (Mugiliformes: Mugilidae).

*Locality*: Bon Secour Bay, Alabama (USA).

*Site in host*: Within scales, in the fins enveloped by lepidotrichia, and in the epidermis between fin rays.

*Prevalence and intensity*: We examined ∼5–20 specimens of each fish species; each individual of each fish species was infected (100% prevalence) with approximately 5–50 cysts.

*Specimens deposited*: USNM 1774172–1774175.

*Representative DNA sequences: 28S* GenBank accession No. PZ513907–PZ513912, *ITS2* GenBank accession No. PZ513851–PZ513856.

*Description (based on 21 encysted specimens, 3 excysted, heat-killed, stained and permanently whole-mounted specimens, and 3 excysted specimens observed alive in temporary wet-mounts*): Cyst spheroid, membranous-like, transparent (Figure 3A), 163–188 (178 ± 8; 21) by 158–196 (171 ± 10; 21), within fish scales and fin rays. Excysted metacercaria, 249–312 (289; 3) in total length, 97–119 (108; 3) in maximum width. Tegument spinose; tegumental spines visible in anterior third of body (Figure 5E). Ventral sucker spheroid, post-equatorial (Figure 5E), 31–44 (36; 3) in total length, 30–39 (36; 3) in maximum width. Oral sucker terminal, infundibular, 76–81 (79; 3) in total length, 97–119 (108; 3) in maximum width, having circumoral spines (Figure 5E); oral sucker spines 27 in number, widest at insertion, tapering posteriad (Figure 5E), 21 (3) in total length, 4 (3) width at insertion. Oesophagus medial (Figure 5E). Pharynx ovoid in outline, 42–47 (44; 3) in total length, 26–28 (27; 3) in maximum width (Figure 5E). Intestinal bifurcation not observed. Ceca extending posteriad parallel to lateral body margins; each cecum opening through a posterolateral pore (anal pore). Excretory vesicle not observed.

#### Adult (Figures 5F–H and 6A–B; Table 3)

*Type host and host reported herein*: American alligator, *Alligator mississippiensis* (Daudin, 1802) (Crocodilia: Alligatoridae).

*Type locality*: Unknown.

*Localities reported herein*: Alabama (USA); Dennis Wildlife Center Fish Hatchery (Santee Cooper Reservoir, Cooper River drainage, South Carolina, USA).

*Site in host*: Intestine.

*Prevalence and intensity*: 3 of 11 (27%) alligators were infected with 1–5 *P. diploporum*.

*Specimens examined: Proctocaecum diploporum* holotype AMNH 875, and paratype AMNH 875A.

*Specimens deposited*: USNM 1774184–1774186.

*Representative DNA sequences: 28S* GenBank accession No. PZ513849, *ITS2* GenBank accession No. PZ513905.

**Figure 6.**
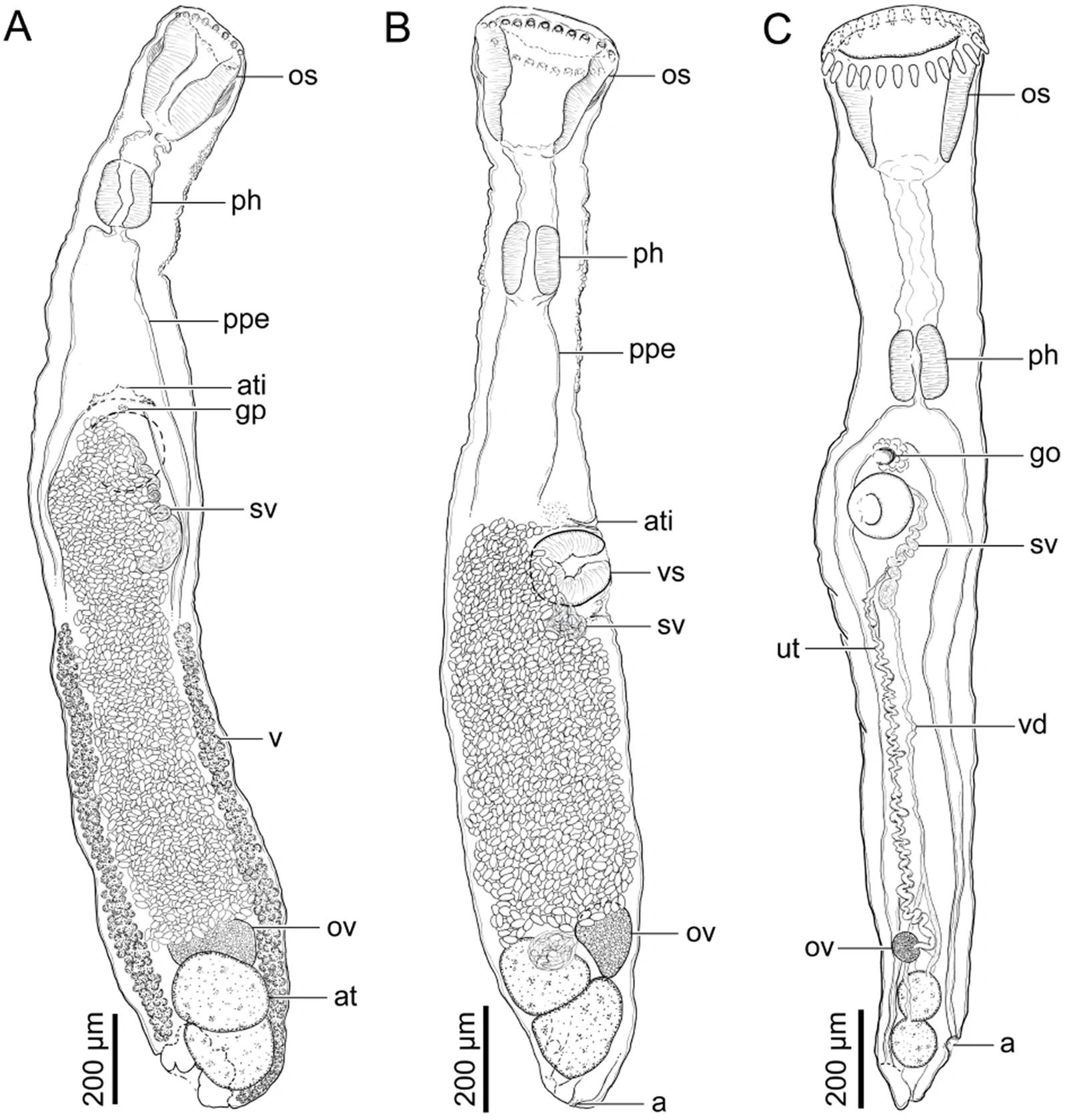
Types of *Proctocaecum diploporum* (Stunkard, 1931) Baugh, 1957 (Digenea: Cryptogonimidae) from American alligator, *Alligator mississippiensis* (Daudin, 1802) (Crocodilia: Alligatoridae) from an indeterminate geographic locality. Scale value aside each bar. (A) Holotype (AMNH Platyhelminthes 875). Dorsal view. (B) Paratype (AMNH Platyhelminthes 875A). Lateral view. (C) Juvenile specimen of *Proctocaecum coronarium* (Cobbold, 1861) Brooks, 1980 (AMNH Platyhelminthes 880). Ventral view. Abbreviations: anal pore (a); anterior tegumental infolding (ati); anterior testis (at); genital pore (gp); gonotyl (go); oral sucker (os); ovary (ov); pharynx (ph); post-pharyngeal oesophagus (ppe); seminal vesicle (sv); seminal vesicle (sv); uterus (ut); ventral sucker (vs); vitellarium (v).

*Supplemental description (based on holotype AMNH 875, paratype AMNH 875A, and 3 heat-killed, stained and permanently whole-mounted adults)*:

Body elongate, rounded posterior end (Figures 5F, 6A and 6B), 10–13 × longer than wide. Forebody 34–43% (39%; 3) of total body length. Hindbody 54–62% (57%; 3) of total body length. Tegument spinose, having 2 tegumental infoldings (Figures 5F, 5G, 6A and 6B); body surface spines big (Figures 5F, 4B and 4D), scale-like, becoming bigger at level of ventral sucker, then becoming smaller and fewer posteriad, extending to near posterior end of body; tegumental infoldings anterior and posterior to ventral sucker (Figures 5F, 5G, 6A and 6B). Ventral sucker spherical, in first quarter of body (Figures 5F, 6A and 6B). Oral sucker terminal, infundibular shaped, having circumoral spines; oral sucker spines forming a collar-like structure (Figures 5F, 6A and 6B), widest at insertion, tapering posteriad (Figure 5F). Oesophagus medial, representing 21–33% (28%; 3) of total body length, comprising pre-pharyngeal, pharyngeal and post-pharyngeal parts (Figures 5F, 6A and 6B). Pre-pharyngeal oesophagus retractile, 12–22% (17%; 3) of total oesophagus length. Pharynx quadrate in outline (Figures 5F, 6A and 6B), surrounding oesophagus, representing 11–20% (14%; 3) of total oesophagus length, followed by short muscular portion, sphincter-like, then followed by post-pharyngeal oesophagus. Post-pharyngeal oesophagus very long, thick-walled, 63–75% (69%; 3) of total oesophagus length (Figures 5F and 6A–B). Intestine bifurcating at level of ventral sucker (Figures 5F and 6A–B), 31–41% (36%; 3) of body length to anterior body end. Ceca slender, extending posteriad parallel to lateral body margins, symmetrical in length and width, each cecum opening through terminal pore (anal pore); anal pores terminal, flanking excretory pore (Figure 5F).

Testes 2, spherical, medial, in last quarter of body, in tandem, contiguous with ovary (Figures 5F, 6A and 6B). Vasa efferentia obscured by dense mass of eggs through its length. Seminal vesicle long, sinuous, extending posterior to ventral sucker (Figures 5F, 5G, 6A and 6B); having convoluted proximal portion, having distal portion enlarged, sac-like (Figures 5F and 6A); prostatic duct short, weakly muscular, followed by ejaculatory duct. Ejaculatory duct short, opening into genital pore through a hermaphroditic duct (Figure 5F and 5G). Genital pore medial, between ventral sucker and anterior tegumental infolding (Figures 5F, 5G, 6A and 6B). Muscular gonotyl absent, but a dense mass of glandular-like cells is present in tegumental infoldings (Figures 5F, 5G, 6A and 6B).

Ovary spheroid (Figures 5F, 6A and 6B); pre-ovarian space 81–85% (84%; 3) of total body length; post-ovarian space 11–13% (12%; 3) of total body length. Oviduct emerging dorsally from ovary. Oviducal seminal receptacle sac-like, postero-dorsal to ovary. Laurer’s canal long, extending posterior to ovary, having a dorsal opening. Oötype, and Mehlis’ gland not observed. Uterus coiling anteriorly between ceca, extending from ovary to common genital pore (Figures 5F, 6A and 6B), dorsal to seminal vesicle, filled with numerous eggs (Figures 5F, 6A and 6B), confluent with hermaphroditic duct. Hermaphroditic duct opening into genital pore (Figures 5F and 5G). Eggs ovoid, having an operculum (Figure 5H). Vitellarium follicular, distributing in 2 bilaterally symmetrical fields, extending from anterior margin of anterior testis to equator of body (Figures 5F and 6A), occupying 38–43% (40%; 3) of total body length; pre-vitellarium space 50–55% (53%; 3) of total body length; post-vitellarium space 5–9% (7%; 3) of total body length. Transverse vitelline ducts emanating from posterior quarter of vitellarium, at level of ovary, forming vitelline reservoir; vitelline reservoir dorsal to ovary, confluent with oviduct (Figure 5F).

Excretory vesicle Y-shaped, ventral, extending from posterior body end to middle portion of pre-pharyngeal oesophagus (Figure 5F), bifurcating posterior to ventral sucker (Figure 5F); excretory pore terminal (Figure 5F).

### Taxonomic remarks for *P. coronarium* and *P. diploporum*

Juveniles of *P. coronarium* and *P. diploporum* were morphologically similar and difficult to differentiate without having high quality, heat-killed and well-stained whole-mounted specimens and observations of live specimens with DIC. The redia were nearly identical. The cercaria and metacercaria of *P. coronarium* are 1.2–1.6 × larger than those of *P. diploporum* (see descriptions above). The adult of *P. coronarium* differ from *P. diploporum* by a combination of having smaller tegumental spines (2–5 long) in the forebody (vs 7–14 long), 24 oral sucker spines (vs 26–27), a short (29–50 long; 9–10% of oesophagus length) post-pharyngeal oesophagus (vs 543–1517 long; 63–75% of oesophagus length), asymmetrical ceca with anal pores at level of posterior testis (vs symmetrical ceca with anal pores in posterior end of body), a muscular gonotyl (vs absent), a vitellarium extending from the ovary to the posterior margin of the ventral sucker (vs extending from posterior testis to middle portion of body), excretory vesicle arms extending anteriad to level of the pharynx (vs extending anteriad to pre-pharyngeal oesophagus), and a dorsal excretory pore in the posterior body end (vs having a postero-terminal excretory pore).

The taxonomic history of *Proctocaecum*, its type species *P. diploporum*, and *P. coronarium* is convoluted and needs clarification. This confusion probably exists because these species are morphologically similar, they infect the same definitive host (the American alligator), their original descriptions contain errors, the type series of *P. coronarium* was lost or never deposited (Brooks and Overstreet, 1977; present study), and the type series for *P. diploporum* includes more than 1 species (see below). Brooks and Overstreet (1977) asserted that Cobbold (1861) probably failed to note the presence of a pharynx and anal pores and that he misinterpreted ‘*darkly-staining* … *parenchymal cells*’ (Brooks and Overstreet, 1977; p. 1020) as vitelline follicles that extend anteriorly to the oesophageal bifurcation in *P. coronarium*. Based on our newly collected specimens and the examination of *P. coronarium* museum vouchers, we agree with Brooks and Overstreet (1977) regarding the presence of a pharynx, anal pores and the extension of the vitellarium from anterior margin of anterior testis to posterior margin of ventral sucker in *P. coronarium.* Anal pores were erroneously omitted in several early descriptions of cryptogonimids. For example, when *Acanthostomum* was proposed by Looss (1899), he described the ceca as blind ending. Later, Khalil (1963) and Moravec (1976, 2001) asserted that Looss (1899) overlooked the anal pores and confirmed their presence in its type species *Acanthostomum spiniceps* (Looss, 1896). Baugh (1957), evidently unaware of the presence of anal pores in *Acanthostomum* Looss, 1899, proposed *Proctocaecum* Baugh, 1957 and designated *P. diploporum* as its type species. Brooks and Overstreet (1977) collected cryptogonimids from American alligators from Florida, Mississippi and Louisiana (USA). They considered *P. diploporum* (as *Acanthostomum diploporus* [Stunkard, 1931] Stunkard, 1938) a junior subjective synonym of *P. coronarium* (as *Acanthostomum coronarium* [Cobbold, 1861] Looss, 1899) stating that specimens from Stunkard’s collection are identical to specimens they identified as *P. coronarium*. Later, in his revision of the Acanthostomidae, Brooks (1980) established the new combination *P. coronarium*, erroneously listed Baugh (1957) as the taxonomic authority, and considered it the type species of *Proctocaecum*. Instead of Baugh (1957), Brooks (1980) is the correct taxonomic revisor for *P. coronarium*.

Based on our newly collected specimens and our study of the type specimens, we accept *P. diploporum* as distinct, therefore *P. diploporum* is the type species of *Proctocaecum* because Baugh (1957) originally designated it (International Commission on Zoological Nomenclature ICZN, 1999; Article 67.4). Brooks and Overstreet (1977) also described *C. pavida* as *Acanthostomum pavidum* Brooks and Overstreet, 1977. *Caimanicola pavida* is morphologically identical to *P. diploporum* because it has large forebody spines (Table 3), 26–28 oral sucker spines, a long post-pharyngeal oesophagus (Table 3), symmetrical ceca with anal pores in the posterior body end, a vitellarium that extends from the posterior testis to the middle portion of the body, and a terminal excretory pore in the posterior body end as well as by lacking a muscular gonotyl. Therefore, we consider *C. pavida* a junior subjective synonym of *P. diploporum*. However, and importantly, future workers should be aware that the type series of *P. diploporum* and the type series of ‘*C. pavida*’ comprises some misidentified specimens. Specifics of these problems and the clarifications are below.

First, the type series of *P. diploporum* comprises 11 slides deposited in the AMNH collection. The holotype is mounted on the same slide as one of the paratypes (AMNH 875 and 875A), which are illustrated herein (Figures 6A and 6B, respectively). These specimens are indeed *P. diploporum* and are distinct from each of the remaining 10 paratypes (on 10 slides) that can only be morphologically diagnosed as *P. coronarium* (AMNH 876–881; AMNH 880 is Figure 6C). We herein identified them as immature specimens of *P. coronarium* because they have a short post-pharyngeal oesophagus, minute tegumental spines, 24 oral sucker spines, asymmetrical cecae, and anal pores at level of the posterior testis (vs the holotype [AMNH 875] and paratype [875A] of *P. diploporum* that have a long post-pharyngeal oesophagus, large tegumental spines, symmetrical ceca and terminal anal pores). We cannot determine the number of oral sucker spines in the holotype AMNH 875 and paratype 875A since they are highly contracted and have evidently lost the oral spines (Figures 6A and 6B). Stunkard (1931) described *P. diploporum* as having 24 oral sucker spines but his drawing shows 26. Additionally, Stunkard’s drawing of *P. diploporum* has a short post-pharyngeal oesophagus (see Figure 1 in Stunkard, 1931) but we observed it to be long (Figure 6A). The misidentified paratypes (AMNH 876–881) should be regarded as vouchers of *P. coronarium*.

Second, the types of *C. pavida* were deposited in the USNM, the HWML and the Natural History Museum, Parasitic Worms Collection (NHMUK; London, UK). The holotype (USNM 74505) is identical to our specimens of *P. diploporum*. Regarding the paratypes, 3 of 26 slides under accession number HWML 20851 and 1 out of 6 slides under HWML 20852 are in fact *P. coronarium*.

#### Designation of a neotype for *P. coronarium*

*Proctocaecum coronarium* was described by Cobbold (1861), and later illustrated (Cobbold, 1864) based on specimens he collected from an American alligator maintained at the Zoological Society of London Menagerie (the London Zoo). He did not designate a holotype, and no name-bearing type material exists to our knowledge: the USNM, AMNH and NHMUK lack a name bearing type for *P. coronarium*. Therefore, the type series of *P. coronarium* is considered lost or never deposited (Brooks and Overstreet, 1977; present study). The absence of a name-baring type has contributed to the long-standing taxonomic confusion involving *P. coronarium, P. diploporum* and *C. pavida* (see Taxonomic Remarks). Designation of a neotype therefore is warranted and necessary to clarify its taxonomic identity and to stabilize nomenclature within *Proctocaecum* (in accordance with International Commission on Zoological Nomenclature ICZN, 1999; Article 75.3). Although the original specimens of *P. coronarium* were collected from a host maintained in captivity in London, the American alligator native range is the southeastern USA. Since *P. coronarium* has been re-collected on several occasions from USA (Brooks and Overstreet, 1977; unpublished vouchers in USNM Invertebrate Zoology Collection; present study), we inferred that Cobbold’s (1861) specimens also came from within the natural range of the host. The neotype designated herein was collected from an American alligator within its native distribution in Alabama (southeastern USA), closely approximating the inferred type locality and host association of the original material.

Based on the careful consideration of the above information, we designate a neotype (an adult specimen) of *P. coronarium* (USNM 1774187). The specimen conforms to Cobbold’s (1861) original description and is consistent with the concept of *P. coronarium* as distinguished from its congeners (see Taxonomic Remarks). The neotype is designated as the name-bearing type to ensure long-term nomenclature stability and accessibility for future study as per the ICZN.

### Histology of metacercarial infections in fish scales

Our histological sections confirmed that the metacercarial cyst resides within the scale, not within the epidermis covering the scale nor within the dermis beneath the relatively transparent scale (Figure 3B). A thin basophilic structure corresponding to the metacercaria cyst wall is clear (Figure 3B). No inflammatory response was observed. The metacercariae was identified as belonging to a species of *Proctocaecum* because it has conspicuous spines in the oral sucker.

### Phylogenetic results

Our *28S* and *ITS2* sequences representing all developmental stages of *P. coronarium* and the adults and metacercariae of *P. diploporum* comprised 1620 bp and 402 bp, respectively. Our *28S* sequences of the cercariae of *P. diploporum* comprised 1303 bp. We herein provide the first nucleotide information for *P. macroclemidis* (voucher USNM 1774212–1774214; see Materials and Methods), its *28S* sequence comprised 1620 bp. GenBank accession numbers are in Table 2. All life history stages were identical to their corresponding morphologically identified adult, except for the cercaria of *P. diploporum* that was 99.9% similar (1 bp different) to its corresponding adult. We regard this as intra-specific variation. Six metacercarial cysts of each size class (see Materials and Methods) were sequenced. We found that the size of the cyst was a reliable objective character to differentiate the 2 species. The large cysts (>240 μm) had identical *28S* sequences and also matched those of the morphologically identified adult of *P. coronarium*. Likewise, the small cysts (<200 μm) had identical *28S* sequences and also matched those of the morphologically identified adult of *P. diploporum*. Remarkably, in some instances the cysts of both species were encysted mm apart and infecting the same individual scale on the same host individual (Figure 3A).

**Figure 7.**
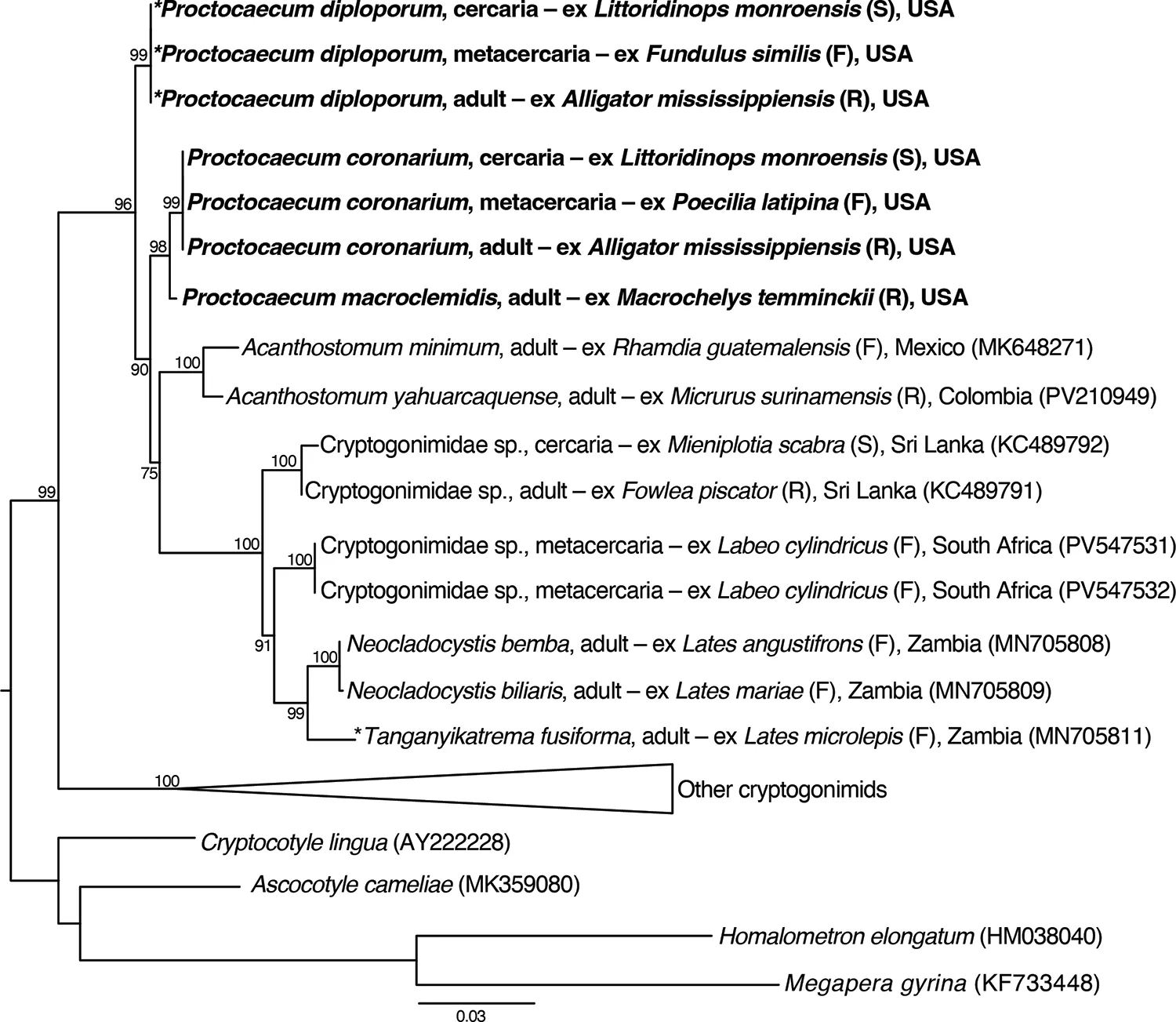
*28S* maximum likelihood phylogenetic analysis inferred tree for Cryptogonimidae. Values aside nodes are ultrafast bootstrap percentage. Scale bar is in substitution per site. GenBank accession numbers are in parenthesis following each taxon. Type species are indicated by asterisk (*). Newly generated sequences are bolded. Figure 7 long description.

Our sequences of *P. coronarium* and *P. diploporum* were 98.6% similar (19–22 bp differences) to each other. Both species were more similar to our sequence of *P. macroclemidis* (*P. coronarium* 99.5%, 8 bp difference; *P. diploporum* 98.9%, 18 bp differences). Our *28S* tree topology (Figure 7) resembled that of Cajiao-Mora et al. (2025) and Martínez-Aquino et al. (2017). However, we included for the first time sequences ascribed to species of *Proctocaecum* and its type species *P. diploporum*. We recovered a paraphyletic *Proctocaecum* with our sequence of *P. diploporum* sister to the clade containing *P. coronarium* + *P. macroclemidis*, that clade being sister to the paraphyletic *Acanthostomum* and several other fish-infecting cryptogonimids. The low support values in our phylogenetic analysis could be the result of low taxon sampling.

## Discussion

We herein describe, for the first time, the life cycle of 2 closely related, sympatric, and morphologically similar reptile-infecting cryptogonimids. Interestingly, both species share the same hosts. We found that metacercariae of both species exhibit low host-specificity, infecting the scales of 7 fish species across Cyprinodontidae, Poeciliidae, Fundulidae, Menidiinae and Mugilidae. The adults, to date, are only known to mature in the American alligator.

The current literature on the life history of reptile-infecting cryptogonimids is scarce. Four studies attempted to characterize, by doing experimental infections, the life cycle of cryptogonimids infecting 3 crocodilians (Ostrowski de Núñez, 1984; Salgado-Maldonado and Aguirre-Macedo, 1991), 1 turtle (Ostrowski de Núñez, 1987), and 1 snake (Roopa and Janardanan, 1998). Ostrowski de Núñez (1984) collected mojarra amarilla, *Caquetaia kraussii* (Steindachner, 1878) (Actinopterygii: Cichlidae), from Lake Valencia (Aragua River), Venezuela, infected with metacercariae of *T. loossi* (as *Acanthostomum loossi*) and *C. marajoara* (as *Acanthostomum marajoara*), in the ‘*skin of the head and the finrays*’. The cysts were fed to 1 spectacled caiman, and 1 Orinoco crocodile, to later collect adults. Similarly to our study herein, Ostrowski de Núñez (1984) documented concurrent infections by 2 cryptogonimids (metacercariae) infecting fish that were initially distinguished by ‘*size, color, and location*’ (site of infection in host) (Ostrowski de Núñez, 1984; p 179). Salgado-Maldonado and Aguirre-Macedo (1991) collected Mayan cichlids, *Mayaheros urophthalmus* (Günther, 1862) (Actinopterygii: Cichlidae) from the Yucatan Peninsula, Mexico, infected with metacercariae of *T. loossi* and fed them to a Morelet’s crocodile in the hopes of recovering adults. Only one type of metacercaria was described in Salgado-Maldonado and Aguirre-Macedo (1991). The cercaria and first intermediate host were not described in either of those studies.

Jayawardena et al. (2013, 2017) used nucleotide information to identify cercariae shed from a thorny tower snail from Siri Lanka as *Acanthostomum burminis* (Bhalerao, 1926) Bhalerao, 1936, a cryptogonimid that infects snakes. They then experimentally exposed Asian common toad tadpoles to assess the effects associated with metacercarial development. However, they provided no morphological identification, deposited no voucher specimen, and did not attempt to obtain adult worms. Consequently, the identity of those specimens is not reliable and should be regarded as Cryptogonimidae sp.

Martínez-Aquino et al. (2017) used nucleotide information and morphology to identify metacercariae of a Mayan cichlid from the Yucatan Peninsula. They identified the metacercariae as *Timoniella* cf. *loossi* and *Acanthostomum* cf. *americanum* because they had spines in the oral sucker but lacked additional morphology. They acknowledge that identifications are not reliable without the adults but that the definitive host could be a species of *Crocodylus* (see Martínez-Aquino et al., 2017). An additional study (see Scholz et al., 1994), on the life cycle of *Oligogonotylus manteri* Watson, 1976, a fish-infecting cryptogonimid in Yucatan, Mexico, reported that the Mayan cichlid is either a second intermediate host (metacercaria infecting gill, fins, body surface, intestine wall) or a definitive host (adults infecting intestinal lumen).

In the Buenos Aires Zoo (Argentina), Ostrowski de Núñez (1987) collected *H. castellanosae* infected with cercariae as well as several freshwater fishes infected with metacercariae of *C. brauni* from a pond holding several species of crocodilians and turtles. Ostrowski de Núñez (1987) experimentally infected a 10 spotted live-bearer with cercaria shed from *H. castellanosae* and fed it to a Hilaire’s side-necked turtle to recover adults. The complete life cycle was successfully elucidated therein. Roopa and Janardanan (1998) collected red-rimmed Melania, *Melanoides tuberculata* (Müller, 1774) (Gastropoda: Thiaridae), from Kerala, India, infected with cercariae of *Acanthostomum burminis* (Bhalerao, 1926) Bhalerao, 1936 and experimentally infected several freshwater fishes, characterized the metacercariae, and then fed the Asiatic water snake with the infected fish to characterize the adults.

We herein provide the first histopathology of a cryptogonimid metacercarial infection. Interestingly, we found that the metacercaria of both *P. diploporum* and *P. coronarium* encysts within the scale itself, i.e., not within the epidermis or dermis. The scale envelopes the metacercarial cyst (Figure 3B). Previous studies report cryptogonimid metacercarial cysts as being beneath the scale or beneath the skin (Ostrowski de Núñez, 1987; Simões et al., 2008; Quintana and Ostrowski de Núñez, 2016). Metacercarial cysts were enveloped by lepidotrichia and observed in the epidermis between fin rays (Lundahl, 1941; Ostrowski de Núñez, 1984, 1987; Simões et al., 2008; Quintana and Ostrowski de Núñez, 2014; Quintana and Ostrowski de Núñez, 2016). Other cryptogonimid metacercariae reportedly infect the buccal cavity, gill, muscle, intestinal wall and liver of fishes (Vélez-Sampedro et al., 2022; Simões et al., 2008; Ostrowski de Núñez et al., 1999; Scholz et al., 1994; Salgado-Maldonado and Aguirre-Macedo, 1991; Maillard, 1973; Cribb, 1986).

Phylogenetically related and sympatric trematodes that use the same invertebrate and vertebrate hosts, like the ones studied here, could be a good model to further explore the mechanisms of digenean speciation and host specificity.

## Table 1 Long description

The table catalogs 13 Proctocaecum species and links each to its type host, type locality, museum type series identifiers, and the original reference. Most species are reported from crocodilians, especially American alligator, Nile crocodile, and saltwater crocodile, with additional records from yacare caiman, gharial, freshwater crocodile, and one turtle host (alligator snapping turtle). Localities span the United States, Sudan, Democratic Republic of the Congo, the Philippines, Indonesia, Brazil, Nepal, and Australia; one entry lists an unknown locality. Several species have no surviving or listed type series, while others provide specific holdings such as AMNH, USNM, MPM, CHIOC, NHMUK, and QM catalog numbers. One species uses a neotype designation (USNM 1774187) for Proctocaecum coronarium. Some entries note that original type material was destroyed and that additional paratypes exist in other museums, so completeness of type series information varies by species.

Navigate back to Table 1.

## Table 2 Long description

The table lists three Cryptogonimidae species and, for each developmental stage sampled, provides USNM museum accession ranges plus GenBank accession numbers for 28S and ITS2 sequences. Proctocaecum diploporum includes redia, cercariae, metacercariae, and adults; redia have only USNM numbers, while cercariae, metacercariae, and adults have both 28S and ITS2 accessions. For diploporum, cercariae have one 28S accession and one ITS2 accession, metacercariae have multiple accessions for both markers, and adults have one accession for each marker. Proctocaecum coronarium also includes all four stages, and each stage has both 28S and ITS2 accessions. Coronarium adults are represented by a neotype plus voucher specimens, with one 28S accession and one ITS2 accession. Proctocaecum macroclemidis is represented only by adults, with one 28S accession and one ITS2 accession. Dashes indicate that no GenBank accession number is provided for that marker and stage.

Navigate back to Table 2.

## Figure 7 Long description

The diagram is a phylogenetic tree representing the maximum likelihood analysis of Cryptogonimidae. The tree is oriented vertically, with branches extending from a common ancestor at the bottom. Each branch represents a taxon, with labels indicating species names, life stages and host information. GenBank accession numbers are in parentheses following each taxon. Type species are marked with an asterisk. Newly generated sequences are bolded. Bootstrap values are shown beside nodes, indicating the support for each branch. The scale bar at the bottom represents substitutions per site. The tree includes various species such as ′Proctoecum diplobulbum′ and ′Proctoecum coronarium′ from different hosts and locations, as well as other genera like ′Acanthostomum′ and ′Neocladocystis′. The tree also includes outgroups such as ′Cryptopygop linga′ and ′Ascocotyle carmelae′.

Navigate back to Figure 7.

## Table 3 Long description

The table compiles morphometric measurements for adult Proctocaecum coronarium and Proctocaecum diploporum from American alligators, comparing multiple sources and localities. Sample sizes range from 3 to 30 in modern studies, while one historical report lists an unspecified number of specimens. In the present study, P. coronarium has a total body length of about 1.7 to 2.5 mm, whereas P. diploporum is larger at about 4.0 to 6.4 mm; a separate report of P. coronarium lists a much longer single value, and the P. diploporum form reported as C. pavida spans a very wide length range. Forebody and hindbody lengths follow the same pattern, with P. diploporum showing notably longer segments than P. coronarium where reported. Body width differs by study and measurement point, but P. diploporum commonly reaches similar or greater maximum widths than P. coronarium, and the C. pavida report includes the broadest widths. Oral and ventral sucker dimensions overlap between species, but P. diploporum tends to have slightly larger ventral sucker measurements in the present study. Circumoral spine counts are close across taxa, with P. coronarium typically reported with 24 and P. diploporum commonly around 26 to 28, while dorsal spine measurements are missing for some sources. Egg sizes are broadly similar between P. coronarium and P. diploporum in the present study, but the C. pavida report lists larger eggs. Several rows are blank or marked as absent in some sources, so comparisons should be interpreted cautiously because not all traits were measured or reported consistently.

Navigate back to Table 3.

## References

[ref1] Anderson GR and Barker SC (1998) Inference of phylogeny and taxonomy within the Didymozoidae (Digenea) from the second internal transcribed spacer (*ITS2*) of ribosomal DNA. *Systematic Parasitology* 41(2), 87–94. 10.1023/A:1006024128098

[ref2] Baugh SC (1957) Contributions to our knowledge of digenetic trematodes. II. *Proceedings of the National Academy of Sciences, India Section B* 26, 295–313.

[ref3] Boschung HTJ and Mayden RL (2004) *Fishes of Alabama*. Washington, D.C: Smithsonian Press.

[ref4] Brooks DR (1980) Revision of the Acanthostominae Poche, 1926 (Digenea: Cryptogonimidae). *Zoological Journal of the Linnean Society* 70(4), 313–382.

[ref5] Brooks DR and Overstreet RM (1977) Acanthostome digeneans from the American alligator in the southeastern United States. *Proceedings of the Biological Society of Washington* 90(4), 1016–1029.

[ref6] Cable RM and Hunnien AV (1942) Studies on the life history of *Siphodera vinaledwardsii* (Linton) (Trematoda: Cryptogonimidae). *Journal of Parasitology* 28(5), 407–422.

[ref7] Cajiao-Mora K, Brule JH, Dutton HR and Bullard SA (2024a) Supplemental description of *Caballerotrema annulatum* (Diesing, 1850) Ostrowski de Núñez and Sattmann, 2002 (Digenea: Caballerotrematidae) from a new host (*Electrophorus* cf. *var*ii) and locality (Amazon River, Colombia) with phylogenetic analysis and emended generic diagnosis. *Journal of Parasitology* 110(4), 276–294. 10.1645/24-9 38982635

[ref8] Cajiao-Mora K, Brule JH, Dutton HR, Caicedo-Portilla JR and Bullard SA (2025) *Acanthostomum yahuarcaquense* n. sp. (Digenea: Cryptogonimidae) infecting the intestine of an aquatic coral snake, *Micrurus surinamensis* (Cuvier, 1817) (Serpentes: Elapidae) from the flooded rainforest habitat of the Yahuarcaca Lake System (Amazon River, Colombia) and phylogenetic analysis of Cryptogonimidae. *International Journal for Parasitology: Parasites and Wildlife* 26, 101055. 10.1016/j.ijppaw.2025.101055 PMC11986244 40226153

[ref9] Cajiao-Mora K, Brule JH, Warren MB, Ksepka SP, Dutton HR and Bullard SA (2024b) *Alobophora sandrae* n. gen. n. sp. (Digenea: Caballerotrematidae) infecting *Arapaima gigas sensu lato* (Osteoglossiformes: Arapaimidae) with a revision of *Caballerotrema*, key to Caballerotrematidae, and updated phylogeny. *Parasite* 31, 55. 10.1051/parasite/2024054 PMC11418392 39311469

[ref10] Catto JB and Amato JFR (1993) Digenetic trematodes (Cryptogonimidae, Acanthostominae) parasites of the caiman, *Caiman crocodilus yacare* (Reptilia, Crocodylia) from the pantanal mato-grossense, Brazil, with the description of a new species. *Memórias Do Instituto Oswaldo Cruz* 88(3), 435–440.

[ref11] Chernomor O, von Haeseler A and Minh BQ (2016) Terrace aware data structures for phylogenomic inference from supermatrices. *Systematic Biology* 65(6), 997–1008. 10.1093/sysbio/syw037 PMC5066062 27121966

[ref12] Cobbold TS (1861) List of entozoa, including pentastomes, from animals dying at the society’s menagerie, between the years 1857-1860 inclusive with descriptions of several new species. *Proceedings of the Zoological Society of London* 8, 117–128.

[ref13] Cobbold TS (1864) *Entozoa: An Introduction to the Study of Helminthology, with Reference, More Particularly, to the Internal Parasites of Man*. Vol. 1. London, UK: Groombridge and Sons. PMC5180556 30163583

[ref14] Cribb TH (1986) The life cycle and morphology of *Stemmatostoma pearsoni*, gen. et sp. nov., with notes on the morphology of *Telogaster opisthorchis* Macfarlane (Digenea, Cryptogonimidae). *Australian Journal of Zoology* 34(2), 279–304. 10.1071/ZO9860279

[ref15] Cribb TH, Adlard RD and Bray RA (1998) A DNA-based demonstration of a three-host life-cycle for the Bivesiculidae (Platyhelminthes: Digenea). *International Journal for Parasitology* 28(11), 1791–1795. 10.1016/s0020-7519(98)00127-1 9846617

[ref16] Dollfus RP (1950) Trematodes collected in the Belgian Congo by Professor Paul Brien (May-August 1937). *Annales du Musee du Congo Belge. C. Zoologie* 1(1), 1–136.

[ref17] El-Darsh HEM and Whitfield PJ (1999) Digenean metacercariae (*Timoniella* spp., *Labratrema minimus* and *Cryptocotyle concava*) from the flounder, *Platichthys flesus*, in the tidal Thames. *Journal of Helminthology* 73(2), 103–113. 10.1017/S0022149X00700289 10526412

[ref18] Gordillo-González D, Ksepka SP, Dutton HR, Cajiao-Mora K, Brule JH, Kuhajda BR, George AL and Bullard SA (2026) *Mycobacterium gordonae* (Actinomycetota: Mycobacteriales: Mycobacteriaceae) infecting captive-reared Laurel Dace, *Chrosomus saylori* (Cypriniformes: Leuciscidae) in North America. *Journal of Fish Diseases* (e70116). 10.1111/jfd.70116 41504069

[ref19] Heard RW, Overstreet RM and Foster JM (2002) Hydrobiid snails (Mollusca: Gastropoda: Rissooidea) from St. Andrew Bay, Florida. *Gulf and Caribbean Research* 14, 13–34. 10.18785/gcr.1401.02

[ref20] Hoang DT, Chernomor O, von Haeseler A, Minh BQ and Vinh LS (2018) UFBoot2: Improving the ultrafast bootstrap approximation. *Molecular Biology and Evolution* 35(2), 518–522. 10.1093/molbev/msx281 PMC5850222 29077904

[ref21] International Commission on Zoological Nomenclature ICZN (1999) *International Code of Zoological Nomenclature*. London, UK: The International Trust for Zoological Nomenclature. 10.3897/zookeys.931.51583 PMC7205856 32405237

[ref22] Jayawardena UA, Rohr JR, Amerasinghe PH, Navaratne AN and Rajakaruna RS (2017) Effects of agrochemicals on disease severity of *Acanthostomum burminis* infections (Digenea: Trematoda) in the Asian common toad, *Duttaphrynus melanostictus*. *BMC Zoology* 2(1), 13. 10.1186/s40850-017-0022-1

[ref23] Jayawardena UA, Tkach VV, Navaratne AN, Amerasinghe PH and Rajakaruna RS (2013) Malformations and mortality in the Asian Common Toad induced by exposure to pleurolophocercous cercariae (Trematoda: Cryptogonimidae). *Parasitology International* 62(3), 246–252. 10.1016/j.parint.2013.01.003 23353759

[ref24] Jithila PJ and Prasadan PK (2022) Distribution and host-specificity of *Acanthostomum burminis* (Digenea: Cryptogonimidae) metacercaria among different freshwater fishes. *Journal of Ichthyology* 62(6), 1196–1200. 10.1134/S0032945222060121

[ref25] Junker K, Brooks DR and Boomker J (2008) *Proctocaecum gairhei* n. sp. (Digenea: Cryptogonimidae: Acanthostominae) from *Gavialis gangeticus* (Gmelin) in Nepal and a revised cladogram of *Proctocaecum* Baugh, 1957. *Systematic Parasitology* 71(3), 229–236. 10.1007/s11230-008-9151-9 18815902

[ref26] Kalyaanamoorthy S, Minh BQ, Wong TKF, von Haeseler A and Jermiin LS (2017) ModelFinder: Fast model selection for accurate phylogenetic estimates. *Nature Methods* 14(6), 587–589. 10.1038/nmeth.4285 PMC5453245 28481363

[ref27] Katoh K and Standley DM (2013) MAFFT multiple sequence alignment software version 7: Improvements in performance and usability. *Molecular Biology and Evolution* 30(4), 772–780. 10.1093/molbev/mst010 PMC3603318 23329690

[ref28] Khalil LF (1963) On *Acanthostomum gymnarchi* (Dollfus, 1950) with notes on the genera *Acanthostomum* looss, 1899, *Atrophecaecum* Bhalerao, 1940, *Gymnatotrema* Morosov, 1955, and *Haplocaecum* Simha, 1958. *Journal of Helminthology* 37(3), 207–214. 10.1017/S0022149X00003795

[ref29] Kmentová N, Bray RA, Koblmüller S, Artois T, De Keyzer ELR, Gelnar M, Vanhove MPM and Georgieva S (2020) Uncharted digenean diversity in Lake Tanganyika: Cryptogonimids (Digenea: Cryptogonimidae) infecting endemic lates perches (Actinopterygii: Latidae). *Parasites and Vectors* 13(1), 1–24. 10.1186/s13071-020-3913-x PMC7195733 32357898

[ref30] Kvach Y, Bryjová A, Sasal P and Winkler HM (2018) The taxonomic and phylogenetic status of digeneans from the genus *Timoniella* (Digenea: Cryptogonimidae) in the Black and Baltic seas. *Journal of Helminthology* 92(5), 596–603. 10.1017/S0022149X1700075X 28974278

[ref31] Lockyer AE, Olson PD and Littlewood DTJ (2003) Utility of complete large and small subunit rRNA genes in resolving the phylogeny of the Neodermata (Platyhelminthes): Implications and a review of the cercomer theory. *Biological Journal of the Linnean Society* 78(2), 155–171. 10.1046/j.1095-8312.2003.00141.x

[ref32] Looss A (1899) Weitere Beiträge zur Kenntniss der Trematoden-Fauna Aegyptens, zugleich Versuch einer natürlichen Gliederung des Genus Distomum Retzius. *Zoologische Jahrbücher* 12, 521–784.

[ref33] Luna LG (1992) *Histopathologic Methods and Color Atlas of Special Stains and Tissue Artifacts*. Gaithersburg, MD: American Histolabs.

[ref34] Lundahl WS (1941) Life history of *Caecincola parvulus* Marshall and Gilbert (Cryptogonimidae, Trematoda) and the development of its excretory system. *Transactions of the American Microscopical Society* 60(4), 461–484. 10.2307/3222767

[ref35] Maillard C (1973) Etude du cycle évolutif du Trématode: *Acanthostomum imbutiforme* (Molin, 1859) Gohar, 1934, parasite de *Morone labrax* (Linné, 1758). *Annales de Parasitologie Humaine et Compare´e* 48(1), 33–46. 10.1051/PARASITE/1973481033 4728601

[ref36] Maillard C (1974) Cycle evolutif de *Timoniella praeteritum* (Looss, 1901) (Trematoda, Acanthostomidae) parasite de *Morone labrax* (Teleostei, Serranidae). *Bulletine de la Societe Zoologique de France* 99(2), 245–257.

[ref37] Martínez-Aquino A, Vidal-martínez VM and Aguirre-Macedo ML (2017) A molecular phylogenetic appraisal of the acanthostomines *Acanthostomum* and *Timoniella* and their position within Cryptogonimidae (Trematoda: Opisthorchioidea). *PeerJ* 5, e4158. 10.7717/peerj.4158 PMC5729820 29250471

[ref38] Miller TL, Bray RA, Goiran C, Justine JL and Cribb TH (2009) *Adlardia novaecaledoniae* n. g. n. sp. (Digenea: Cryptogonimidae) from the fork-tailed threadfin bream *Nemipterus furcosus* (Val.) (Perciformes: Nemipteridae) off New Caledonia. *Systematic Parasitology* 73(2), 151–160. 10.1007/s11230-009-9187-5 19424793

[ref39] Miller TL and Cribb TH (2007) Coevolution of *Retrovarium* n. gen. (Digenea: Cryptogonimidae) in Lutjanidae and Haemulidae (Perciformes) in the Indo-West Pacific. *International Journal for Parasitology* 37(8-9), 1023–1045. 10.1016/j.ijpara.2007.01.006 17362968

[ref40] Miller TL and Cribb TH (2008a) Eight new species of *Siphoderina* Manter, 1934 (Digenea, Cryptogonimidae) infecting Lutjanidae and Haemulidae (Perciformes) off Australia. *Acta Parasitologica* 53(4), 344–364. 10.2478/s11686-008-0053-4

[ref41] Miller TL and Cribb TH (2008b) Family Cryptogonimidae Ward, 1917. In Bray RA and Gibson DI (eds.), *Keys to the Trematoda*. Wallingford, UK: CAB International, 51–112.

[ref42] Miller TL and Cribb TH (2009) *Gynichthys diakidnus* n. g. n. sp. (Digenea: Cryptogonimidae) from the grunt *Plectorhinchus gibbosus* (Lacépède, 1802) (Perciformes: Haemulidae) off the Great Barrier Reef, Australia. *Systematic Parasitology* 74(2), 103–112. 10.1007/s11230-009-9194-6 19731094

[ref43] Miller TL and Cribb TH (2013) Dramatic phenotypic plasticity within species of *Siphomutabilus* n. g. (Digenea: Cryptogonimidae) from Indo-Pacific caesionines (Perciformes: Lutjanidae). *Systematic Parasitology* 86(2), 101–112. 10.1007/s11230-013-9436-5 24048744

[ref44] Montes MM, Barneche J, Gonzalez TA, Cavallo G, Shimabukuro MI, Moncada M and Martorelli SR (2025) The history continues: From *Steringotrema microacetabularis* (Fellodistomidae) to *Bacciger microacetabularis* (Baccigeridae) genetic evidence reveals a new genus of Cryptogonimidae. *Journal of Helminthology* 99, e46. 10.1017/S0022149X25000239 40062781

[ref45] Moravec F (1976) On two acanthostomatid trematodes, *Acanthostomum spiniceps* (Looss, 1896) and *A. absconditum* (Looss, 1901), from African bagrid fishes. *Folia Parasitologica* 23(3), 201–206. 1010489

[ref46] Moravec F (2001) Some helminth parasites from Morelet’s crocodile, *Crocodylus moreletii*, from Yucatan, Mexico. *Folia Parasitologica* 48(1), 47–62. 10.14411/fp.2001.008 11266135

[ref47] Nguyen LT, Schmidt HA, von Haeseler A and Minh BQ (2015) IQ-TREE: A fast and effective stochastic algorithm for estimating maximum-likelihood phylogenies. *Molecular Biology and Evolution* 32(1), 268–274. 10.1093/molbev/msu300 PMC4271533 25371430

[ref48] Odhner T (1902) Trematoden aus Reptilien nebst allgemeinen systematischen Bemerkungen. *Öfversigt Af Kongliga Vetenskaps-Akademiens Förhandlingar* 59(1), 19–45.

[ref49] Ostrowski de Núñez M (1984) Beiträge zur Gattung *Acanthostomum* (Trematoda, Acanthostomatidae) und zu den Entwicklungszyklen von *A. marajoarum* (Freitas & Lent, 1938) und *A. loossi* (Perez Vigueras, 1957) in Venezuela. *Mitteilungen Aus Dem Zoologischen Museum in Berlin* 60(2), 179–201.

[ref50] Ostrowski de Núñez M (1987) Der entwicklungszyklus von *Acanthostromun brauni* Mañé Garzón und Gil, 1961 (Trematoda, Acanthostomatidae). *Zoologischer Anzeiger* 218(5–6), 273–286.

[ref51] Ostrowski de Núñez M and Gil de Pertierra A (1991) The life history of *Acanthostomum gnerii* Szidat, 1954 (Trematoda: Acanthostomatidae) from the Catfish *Rhmadia sapo* in Argentina. *Zoologischer Anzeiger* 277, 58–71.

[ref52] Ostrowski de Núñez M, Semenas L, Brugni N, Viozzi G and Flores V (1999) Redescription of *Acanthostomoides apophalliformis* (Trematoda, Acanthostomidae) from *Percichthys trucha* (Pisces, Percichthyidae) with notes on its life cycle in Patagonia, Argentina. *Acta Parasitologica* 44(4), 222–228.

[ref53] Quintana MG and Ostrowski de Núñez M (2014) The life cycle of *Pseudosellacotyla lutzi* (Digenea: Cryptogonimidae), in *Aylacostoma chloroticum* (Prosobranchia: Thiaridae), and *Hoplias malabaricus* (Characiformes: Erythrinidae), in Argentina. *Journal of Parasitology* 100(6), 805–811. 10.1645/13-379.1 24945992

[ref54] Quintana MG and Ostrowski de Núñez M (2016) The life cycle of *Neocladocystis intestinalis* (Vaz, 1932) (Digenea: Cryptogonimidae), in *Aylacostoma chloroticum* (Prosobranchia: Thiaridae), and *Salminus brasiliensis* (Characiformes: Characidae), in Argentina. *Parasitology Research* 115(7), 2589–2595. 10.1007/s00436-016-5004-7 26984207

[ref55] Rambaut A, Suchard MA, Xie D and Drummond AJ (2018) FigTree v1.4.4. Available at: http://tree.bio.ed.ac.uk/software/figtree (accessed 10 November 2025).

[ref56] Roopa TM and Janardanan KP (1998) The life cycle of *Acanthostomum burminis*. *Acta Parasitologica* 43(4), 189–193.

[ref57] Salgado-Maldonado G and Aguirre-Macedo L (1991) Metacercarias parasitas de *Cichlasoma urophthalmus* (Cichilidae*) Pelaezia loossi* n. comb. y *Phagicola angrense* con descripción de adultos recuperados experimentalmente. *Anales Del Instituto de Biológia de la Universidad Nacional Autónoma de México Serie Zoología* 62(3), 391–407.

[ref58] Scholz T, Lavadores JIP, Vargas VJ, Mendoza FEF, Rodriguez CR and Vivas RC (1994) Life cycle of *Oligogonotylus manteri* (Digenea: Cryptogonimidae), a parasite of cichlid fishes in southern Mexico. *Journal of the Helminthological Society of Washington* 61(12), 190–199.

[ref59] Simões SBE, Das Neves RFC and Santos CP (2008) Life history of *Acanthocollaritrema umbilicatum* trazvassos, freitas and bührnheim, 1965 (Digenea: Cryptogonimidae). *Parasitology Research* 103(3), 523–528. 10.1007/s00436-008-1000-x 18500539

[ref60] Stunkard HW (1931) Further observations on the occurrence of anal openings in digenetic trematodes. *Zeitschrift Fur Parasitenkunde* 3(4), 713–725. 10.1007/BF02146553

[ref61] Tkach VV and Snyder SD (2010) *Proctocaecum blairi* sp. nov. (Digenea, Cryptogonimidae) from the freshwater crocodile, *Crocodylus johnstoni*, in Northern Territory, Australia. *Acta Parasitologica* 55(3), 240–244. 10.2478/s11686-010-0041-3

[ref62] Tkach VV and Snyder SD (2003) *Acanthostomum macroclemidis* n. sp. (Digenea: Cryptogonimidae: Acanthostominae) from the alligator snapping turtle, *Macroclemys temmincki*. *Journal of Parasitology* 89(1), 159–167. 10.1645/0022-3395(2003)089[0159:AMNSDC]2.0.CO;2 12659321

[ref63] Truong TN, Warren MB, Ksepka SP, Curran SS and Bullard SA (2021) *Posthovitellinum psiloterminae* n. gen. n. sp. (Digenea: Lissorchiidae) infecting the intestine of *Cyclocheilos enoplos* (Cypriniformes: Cyprinidae) in the Mekong River, Vietnam. *Journal of Parasitology* 107(3), 431–435. 10.1645/20-77 34077518

[ref64] Tubangui MA and Masiluñgan VA (1936) Trematode parasites of Philippine vertebrates. VIII. Flukes from a cobra and a crocodile. *Philippine Journal of Science* 60(3), 255–266.

[ref65] Vélez-Sampedro V, Uruburu M and Lenis C (2022) Morphological, molecular, and life cycle study of a new species of *Oligogonotylus* Watson, 1976 (Digenea, Cryptogonimidae) from Colombia. *Zookeys* 1115, 169–186. 10.3897/zookeys.1115.75538 PMC9848881 36761075

[ref66] Warren MB and Bullard SA (2019) First elucidation of a blood fluke (*Electrovermis zappum* n. gen., n. sp.) life cycle including a chondrichthyan or bivalve. *International Journal for Parasitology: Parasites and Wildlife* 10, 170–183. 10.1016/j.ijppaw.2019.06.008 PMC6812027 31667079

[ref67] Yamaguti S (1954) Parasitic worms mainly from Celebes Part 4. Trematodes of reptiles and birds. *Acta Medica Okayama* 8(4), 329–340.

[ref68] Yong RQY, Martin SB and Smit NJ (2023) A new species of *Siphoderina* Manter, 1934 (Digenea: Cryptogonimidae) infecting the Dory Snapper *Lutjanus fulviflamma* (Teleostei: Lutjanidae) from the east coast of South Africa. *Systematic Parasitology* 100(6), 673–686. 10.1007/s11230-023-10116-1 PMC10613151 37845589

[ref69] Zhan TF and Yang ML (1993) Two new genera and species of trematodes from *Andrias davidianus* in Sichuan Province, China (Digenea: Cryptogonimidae). *Acta Zootaxonomica Sinica* 18(3), 270–277.

